# Global Transcriptome Analysis and Identification of Differentially Expressed Genes in Strawberry after Preharvest Application of Benzothiadiazole and Chitosan

**DOI:** 10.3389/fpls.2017.00235

**Published:** 2017-02-24

**Authors:** Lucia Landi, Rita M. De Miccolis Angelini, Stefania Pollastro, Erica Feliziani, Franco Faretra, Gianfranco Romanazzi

**Affiliations:** ^1^Department of Agricultural, Food and Environmental Sciences, Marche Polytechnic UniversityAncona, Italy; ^2^Department of Soil, Plant and Food Sciences, University of Bari ‘Aldo Moro’Bari, Italy

**Keywords:** *Fragaria × ananassa*, gene expression profiling, heat shock proteins, field treatments, photosynthesis, resistance inducers, systemic acquired resistance (SAR), storage proteins

## Abstract

The use of resistance inducers is a novel strategy to elicit defense responses in strawberry fruit to protect against preharvest and postharvest decay. However, the mechanisms behind the specific resistance inducers are not completely understood. Here, global transcriptional changes in strawberry fruit were investigated using RNA-Seq technology. Preharvest, benzothiadiazole (BTH) and chitosan were applied to the plant canopy, and the fruit were harvested at 6, 12, and 24 h post-treatment. Overall, 5,062 and 5,210 differentially expressed genes (fold change ≥ 2) were identified in these fruits under the BTH and chitosan treatments, respectively, as compared to the control expression. About 80% of these genes were differentially expressed by both elicitors. Comprehensive functional enrichment analysis highlighted different gene modulation over time for transcripts associated with photosynthesis and heat-shock proteins, according to elicitor. Up-regulation of genes associated with reprogramming of protein metabolism was observed in fruit treated with both elicitors, which led to increased storage proteins. Several genes associated with the plant immune system, hormone metabolism, systemic acquired resistance, and biotic and abiotic stresses were differentially expressed in treated versus untreated plants. The RNA-Seq output was confirmed using RT-qPCR for 12 selected genes. This study demonstrates that these two elicitors affect cell networks associated with plant defenses in different ways, and suggests a role for chloroplasts as the primary target in this modulation of the plant defense responses, which actively communicate these signals through changes in redox status. The genes identified in this study represent markers to better elucidate plant/pathogen/resistance-inducer interactions, and to plan novel sustainable disease management strategies.

## Introduction

The need to discover alternative crop protection strategies that can be used to improve food safety and security, as well as for maintaining human health, has been the target of many investigations in recent years (Romanazzi et al., 2012; Burketova et al., 2015). In particular, studies have increasingly targeted exogenous molecules that induce defense responses (Walters et al., 2013). In this context, investigations on how non-toxic products can control plant diseases through activation of plant defense responses are fascinating. The effectiveness of compounds that have been described as ‘resistance inducers’ has been tested according to different crop protection strategies. These have shown encouraging results for their use as alternatives to traditional fungicides (Burketova et al., 2015; Oliveira et al., 2016). In particular, the effectiveness of alternative compounds in disease control has been tested in strawberry (*Fragaria × ananassa*), a perishable small fruit crop of great importance throughout the world, but which easily undergoes fungus-mediated preharvest and postharvest decay (Hemelrijcka et al., 2010).

Often the terms ‘resistance inducer’ or ‘elicitor’ have been used for molecules that can protect plants from diseases through induction of their defense mechanisms (Mandal et al., 2013; Walters et al., 2013). This inducible immunity is based on external recognition of ‘non-self’ signals, and notably for pathways of microbe/pathogen-associated molecular pattern (MAMP/PAMP)-triggered immunity (PTI) and effector-triggered immunity (ETI) (Jones and Dangl, 2006; Dodds and Rathjen, 2010). PTI is initiated in plants when PAMPs are recognized by pattern-recognition receptors. In contrast, ETI is induced by recognition of pathogen avirulence effectors by the host disease-resistance (R) proteins. This can lead to rapid and robust responses that are often associated with programmed cell death via the hypersensitive response, and with systemic acquired resistance (SAR) in the host (Ryals et al., 1994; Dodds and Rathjen, 2010). ETI, basal defense, and PTI act through a common set of signaling components, which include multiple regulatory proteins, reactive oxygen species (ROS), Ca^2+^ signaling, and the phytohormones salicylic acid (SA), ethylene (ET), and jasmonic acid (JA) (Spanu, 2012). These signaling events modulate transcription factor (TF) activities that can lead to massive transcriptional reprogramming. This, in turn, results in accumulation of different enzymes and stress-specific metabolites, such as pathogenesis-related (PR) proteins, hydrolytic enzymes, peroxidases and phytoalexins, and deposition of lignin and callose (Lamb and Dixon, 1997; Oliveira et al., 2016). Induction of resistance in host tissues can also lead to production of beneficial antimicrobial compounds (Romanazzi et al., 2016). The onset of PTI and ETI from infected loci often triggers induced resistance in distal tissues that can confer resistance against a broad spectrum of pathogens (Pieterse et al., 2014). SAR is frequently associated with increased levels of SA and coordinated activation of PR genes, which can promote one or more long-distance signals that enhance the defensive capacity of the plant (Fu and Dong, 2013).

Systemic resistance can also be induced by beneficial microbes that are normally associated with plant rot, which is known as induced systemic resistance (ISR), a process that is usually SA and PR-protein independent. During SAR and ISR responses, plants can obtain systemic resistance against different classes of pathogens for several days (Heil and Bostock, 2002). These compelling features of SAR as a defense response are the basis of the induced resistance concepts that underlie the application of specific elicitors.

In strawberry, many non-toxic compounds have been effective in studies carried out with postharvest application, including benzothiadiazole (BTH; also known as acibenzolar-*S*-methyl) and chitosan (Cao et al., 2011; Romanazzi et al., 2013). This also applies to their spraying before harvest in plastic tunnels (Terry and Joyce, 2000) and in the open field (Feliziani et al., 2015). BTH is a light-insensitive analog of SA that can activate defense responses that lead to SAR, and it is an efficient broad-spectrum resistance inducer against bacterial, fungal, and viral diseases in different monocot and dicot crops (Walters et al., 2013). Chitosan is a deacetylated derivative of chitin that is derived from *N*-acetylglucosamine units linked by β-1,4-glycosidic bonds; it is produced by chitin deacetylases, and it occurs naturally as a polysaccharide (Bautista-Baños et al., 2006; Romanazzi et al., 2017). Chitosan treatment produces a coating on the surface of fruit that reduces the gas exchange, which slows down respiration and ripening of the fruit (Romanazzi et al., 2009). The ability of BTH and chitosan to induce gene expression and enzyme activity has been tested in several crops. BTH spraying induces SAR genes that encode PR proteins, and the chitinase, glucanase, and ROS scavenger enzymes (Görlach et al., 1996; Ren et al., 2012; Zhu et al., 2016). Moreover, chitosan can induce plant defense enzymes and synthesis of secondary metabolites in several plant species, such as polyphenolic compounds, lignin, flavonoids, and phytoalexins (Coqueiro et al., 2015; Malerba and Cerana, 2016).

In strawberry fruit, BTH and chitosan elicit defenses by increasing enzyme activities and the expression of specific genes (Cao et al., 2011; Landi et al., 2014). However, a global analysis of the transcriptome responses associated with these elicitor compounds has not yet been performed.

RNA sequencing (RNA-Seq) analysis is a powerful tool to study transcriptomes (Trapnell et al., 2010; AbuQamar et al., 2016). This large-scale analytical approach of gene expression can be crucial to determine the effects of elicitors on plant metabolism. Therefore, here, RNA samples from fruits of strawberry plants treated preharvest with BTH and chitosan were analyzed at 6, 12, and 24 h posttreatment (hpt), and transcript abundances were determined to characterize changes in gene expression patterns.

## Materials and Methods

### Treatments of Strawberry Plants with Resistance Inducers

The treatments were carried out in May 2014, during fruit ripening, on the strawberry cultivar ‘Alba’ (*Fragaria* ×*ananassa;* 2*n* = 8*x* = 56) grown under a high tunnel in an organic orchard in central-eastern Italy (Corridonia; 43°31′60″N, 13°22′60″E), as reported by Feliziani et al. (2015). The treatments were performed by spraying the canopy of the strawberry plants with the elicitors BTH (0.02% w/v; Bion, Syngenta, Milan, Italy) and chitosan (1% w/v; Chito Plant; ChiPro GmbH, Bremen, Germany), as the commercial products suspended in distilled water. Plants sprayed with distilled water were used as the controls.

A randomized block design was used, with each plot 6.5 m in length, which corresponded to ∼45 plants per plot. The plots were divided from each other by 0.5 m of untreated plants. The treatments were carried out by spraying the canopy with a volume equivalent to 1,000 L/ha, using a motorized backpack sprayer (GX 25, 25 cc, 0.81 kW; Honda, Tokyo, Japan). The treatments were performed at 7.30 am, and the ripe fruits were sampled at 6, 12, and 24 hpt. For each sampling time and treatment, three replicated samples of 200 uniform fruits were collected from both the treated and control plants. The samples were immediately frozen in dry ice and stored at -80°C until processed.

### RNA Isolation, RNA-Seq Library Preparation, and Sequencing

For the RNA-Seq analysis, the fruit were ground using an homogeniser (Ultra-Turrax T25; Janke and Kunkel IKA-Labortechnik, Staufen, Germany), and the total RNA was extracted from 1 g of the frozen-powder homogenate, according to Landi et al. (2014). The RNA quantity and quality were determined using a Nanodrop 2000 (Thermo Fisher Scientific Inc., Wilmington, DE, USA) and a bioanalyzer (model 2100; Agilent Technologies, Santa Clara, CA, USA). cDNA libraries were prepared from 4 μg total RNA using TruSeq RNA Sample Preparation kits v2 (Illumina, Inc., San Diego, CA, USA), and validated according to the Illumina low-throughput protocol. After normalization, the cDNA libraries were pooled for multiplexing, before loading onto a flow cell (five samples per lane). The hybridization and cluster generation were performed on a cBot System using TruSeq SR Cluster kits v3 (Illumina). The sequencing was performed with an Illumina HiScanSQ platform, using TruSeq SBS kits v3 (Illumina) to obtain single reads 50 nt in length. The indexed raw sequencing reads from each library were de-multiplexed using the CASAVA v1.8 software (Illumina).

### RNA-Seq Data Analysis

The quality of the raw sequence reads was checked using FastX-tools^1^ (Blankenberg et al., 2010). The filtered reads from each sample were then separately aligned using CLC genomics Workbench v.7.0.3 (CLCbio, Qiagen, Aarhus N, Denmark) on the *Fragaria vesca* subsp. *vesca* genome (2*n* = 2*x* = 14) (FraVesHawaii_1.0, annotation release 101)^2^, used as a reference. Default mapping parameters were used for RNA-Seq analysis, to estimate the abundance of 31,380 gene transcripts, measured as reads per kilobase per million mapped reads (RPKM) (Trapnell et al., 2010). A RPKM ≥ 0.5 was used as the cut-off for gene expression (Kang et al., 2013; Hollender et al., 2014).

The differential expression analysis was carried out through comparisons of RPKM expression values following the treatments with BTH and chitosan, and the water-sprayed control, at each time point. Genes with fold-change (FC) ≥ 2 for at least one of the sampling times were considered as differentially expressed genes (DEGs) and were submitted to functional analysis. The expression profiles of the DEGs at different time points were analyzed by hierarchical clustering and heat maps of FC after BTH and chitosan treatments, as compared to the water control (T-MeV 4.9.0 software; Howe et al., 2011).

### Functional Analysis

For the Blastx and Gene Ontology (GO) analyses, the gene transcripts of *F. vesca* were loaded onto Blast2GO v.2.8^3^ and separated using the GO vocabulary^4^. The ontology annotations were then refined using InterProScan (Conesa et al., 2005). The DEGs were then assigned to the GO categories for annotation and description of their biological functions (Ashburner et al., 2000).

Fisher’s exact tests were used to identify significantly enriched GO terms (false discovery rate [FDR], corrected *P*-value ≤ 0.05). Pathway analysis was performed using the Kyoto Encyclopedia of Genes and Genomes (KEGG)^5^ functions of the Blast2GO platform. Significantly enriched KEGG pathways were identified with KO-based annotation system (KOBAS) 2.0 (Xie et al., 2011), using the *p*-value. In addition, PAGEMAN (Usadel et al., 2006) in the MapMan 3.5.1R2 (Thimm et al., 2004) software package^6^ was used to explore the functional classes. PAGEMAN clusters data points of each up-regulated and down-regulated sequence in hierarchical classification of the genes (i.e., bins); each bin that showed FC ≥ 2.0 was tested for over and under representation using bin-wise Wilcoxon tests. Furthermore, the resulting *p*-values of 0.05 were adjusted according to Benjamini and Hochberg corrections for multiple tests. In this test, the median log_2_ ratios for all of the genes in a particular MapMan annotation bin were compared with the median log_2_ ratios of all of the other MapMan bins, using *F. vesca* (Fvesca_226) mapping.

### Real-Time Reverse-Transcription Quantitative PCR Validation of the DEGs

To verify the data from the RNA-Seq analysis, 12 representative DEGs related to photosynthesis, heat-shock proteins (HSPs), ROS-scavenger metabolism, SAR signaling, storage proteins and secondary metabolism, were selected for real time reverse-transcription quantitative (RT-qPCR) analysis. Specific primers were designed using the Primer3 software^7^ (Supplementary Table S1). First-strand cDNA was synthesized using iScript TM cDNA synthesis kits (Bio-Rad Laboratories, Hercules, CA, USA) from the RNA samples obtained from the strawberry fruits, as previously described. RT-qPCR reactions were carried out in duplicate in a total volume of 16 μL, which contained 7 μL diluted (1:5) cDNA, 0.25 μM of each primer, and 8 μL SsoFast EvaGreen Supermix, in a CFX Connect Real Time Detection System (Bio-Rad Laboratories). The cycling conditions were as follows: 4 min denaturation at 98°C, followed by 40 cycles at 98°C for 15 s, and 60°C for 40 s. Melting curve analysis was performed over the range of 65 to 98°C. Relative changes in gene expression were determined using the 2^-ΔΔCt^ method (Livak and Schmittgen, 2001) with *18S* and *ACTIN* as reference genes (Landi et al., 2014).

## Results

### RNA-Seq Analysis

RNA-Seq data were generated from strawberry fruits sampled 6, 12, and 24 hpt from plants treated preharvest with BTH and chitosan, as compared to the control. After removing low-quality reads, 7 million to 16 million reads per sample were mapped against the reference genome. An average of 95.6% of the filtered short reads (50 nt in length) mapped to the *F. vesca* genome, and an average of 91.5% of these mapped to coding DNA sequences (**Table 1**). Similar data in terms of percentages of read mapping were obtained when the *Fragaria* ×*ananassa* (octoploid) reference genome (FANhybrid r1.2, v1.0) ^8^ was used as reference, with a high number of annotated fragmented transcripts (data not shown). The reads that mapped on coding DNA sequences were used in the subsequent analyses.

**Table 1:** Summary of the read numbers aligned onto the *Fragaria vesca* reference genome.

Experimental condition	Sampling time (hpt)	Total reads (*n*)	Total mapped reads [*n*, (%)]	Mapped reads (*n*)		Differentially expressed genes; FC ≥ 2 (*n*)	
				Unique matches	Multi-position matches	Up-regulated	Down-regulated
BTH	6	14,963,460	14,320,161 (95.7)	13,777,675	542,486	1,064	672
	12	16,837,111	16,095,424 (95.6)	15,481,150	614,274	1,055	814
	24	7,710,040	7,378,442 (95.7)	7,066,148	312,294	1,815	970
Chitosan	6	15,458,121	14,773,256 (95.6)	14,177,017	596,239	1,121	629
	12	7,051,220	6,733,176 (95.5)	6,268,943	464,233	1,313	1,218
	24	7,701,160	7,361,530 (95.6)	7,061,131	300,399	1,255	998
Control (water)	6	15,060,037	14,401,449 (95.6)	13,468,960	932,489	–	–
	12	15,567,712	14,885,852 (95.6)	14,344,917	540,935	–	–
	24	7,850,172	7,511,895 (95.7)	7,132,727	379,168	–	–

### Differential Gene Expression

**FIGURE 1:**
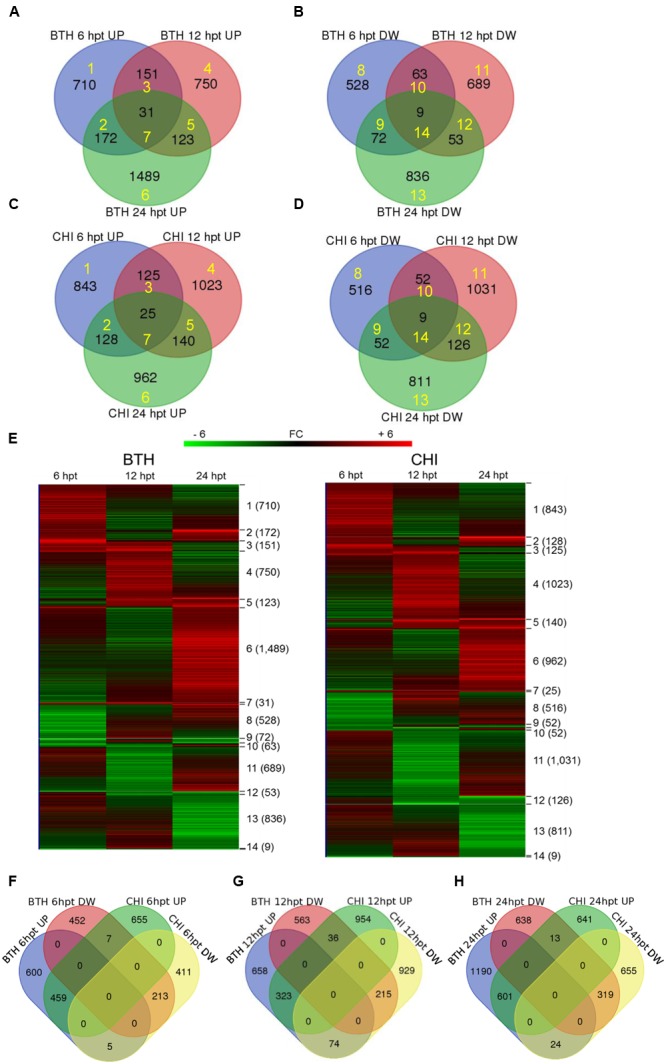
**Venn diagrams showing the overlap of the differentially expressed genes (DEGs; fold change; | FC | ≥ 2) that were up-regulated (UP) and down-regulated (DW) in the strawberry fruit at 6, 12, and 24 h post-treatment (hpt) by BTH (A,B)** and chitosan (CHI; **C,D**), and the related heat maps **(E)**. Venn diagrams comparing the response to BTH and chitosan at different times after the treatments (hpt) **(F–H)**. Yellow numbers represent sets identified by Venn diagrams and analyzed by hierarchical clustering. Software (http://bioinformatics.psb.ugent.be/webtools/Venn/) was used for the Venn diagram.

Across all of the times tested, the data for BTH and chitosan versus control produced 5,062 and 5,210 transcripts, respectively, of DEGs (i.e., | FC |≥ 2). Following their detection, these DEGs were submitted to clustering and functional analysis. The numbers of DEGs associated with the BTH and chitosan treatments for each time point are reported in **Table 1**; Supplementary Table S2. Among the up-regulated genes, 0.9 and 0.77% of the genes associated with BTH and chitosan, respectively, were differentially expressed at all time points. Among the down-regulated genes, 0.4 and 0.34% of the genes associated with BTH and chitosan, respectively, were differentially expressed at all time points (**Figures 1A–D**, sets 7, 14).

Most of the DEGs were modulated only at single sampling times (sets 1, 4, 6, 8, 11, and 13). The expression profiles of the DEG sets are reported in **Figure 1E**. At 24 hpt, BTH induced up-regulation of a larger number of genes than chitosan (set 6), whereas at 12 hpt more genes were up-regulated (set 4) or down-regulated (set 11) by chitosan than BTH. Comparing across the two elicitors at 6, 12, and 24 hpt, 21.0, 13.6, and 19.6%, respectively, of the genes were shared among the up-regulated genes; likewise, 16.4, 10.6, and 16.2%, respectively, were shared among the down-regulated genes (**Figures 1F–H**). The DEGs highly regulated by the elicitors increased from 6 to 24 hpt. In this regard, for BTH, the proportions of up-regulated and down-regulated transcripts with | FC |≥ 8 at 6 hpt were 4.6 and 3.6%, at 12 hpt 5.1 and 1.5%, and at 24 hpt 8.9 and 6.5%, respectively. For chitosan, at 6 hpt these were 2.5 and 3.0%, at 12 hpt 4.7 and 4.9%, and at 24 hpt 8.8 and 6.9%, respectively (Supplementary Table S2).

### Functional Analysis

**FIGURE 2:**
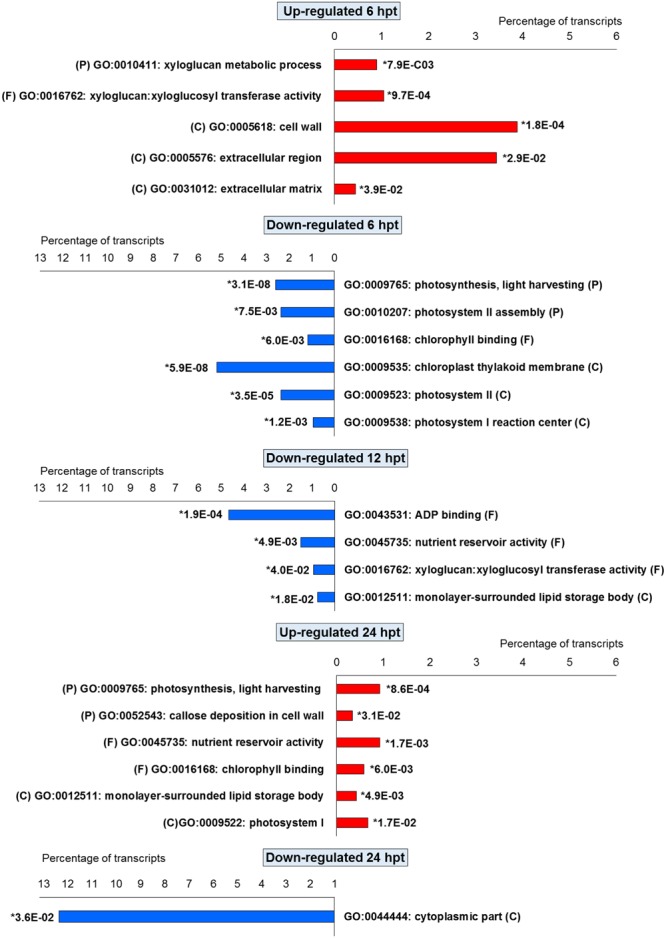
**Gene Ontology terms significantly enriched (^∗^false discovery rate, ≤ 0.05) among the DEGs (fold change; | FC | ≥ 2) identified in strawberry fruit following treatment with BTH at 6, 12, and 24 h post-treatment (hpt).** (P) biological processes; (F) molecular functions; and (C) cellular components.

**FIGURE 3:**
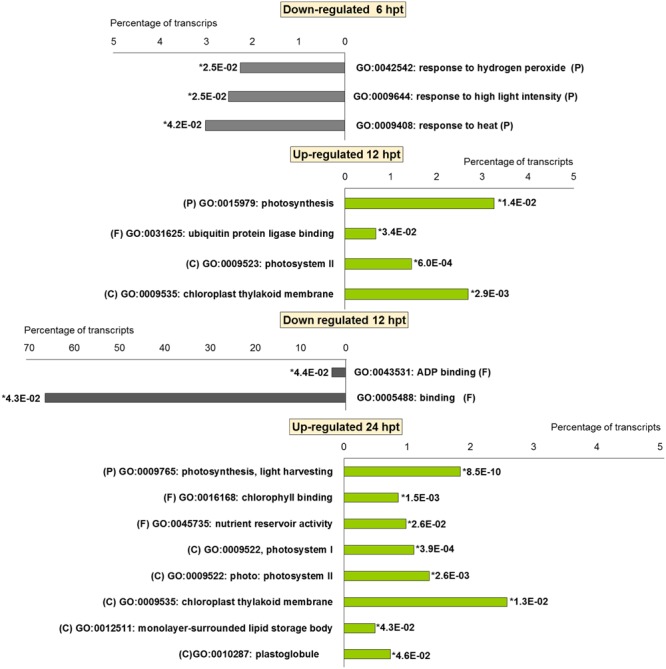
**Gene Ontology terms significantly enriched (^∗^false discovery rate, ≤ 0.05) among the DEGs (fold change; | FC | ≥ 2) in strawberry fruit following treatment with chitosan at 6, 12, and 24 hpt.** (P) biological processes; (F) molecular functions; and (C) cellular components.

For BTH, 111 up-regulated DEGs were significantly enriched in 11 GO terms, and 178 down-regulated DEGs in 11 GO terms. For chitosan, 153 up-regulated DEGs were significantly enriched in 12 GO terms, and 554 down-regulated genes DEGs in 5 GO terms (**Figures 2** and **3**; Supplementary Tables S2 and S3).

For both the BTH-treated and chitosan-treated strawberries, there was differential modulation on the time of the light phase of the photosynthetic process, the reservoir of nutrients, and the lipid-metabolism-associated storage organelles. In contrast, the influences on cell-wall, extracellular matrix, and extracellular region were only associated with BTH, while the response to heat, hydrogen peroxide and high light intensity were affected chitosan (**Figures 2** and **3**; Supplementary Tables S2 and S3).

**FIGURE 4:**
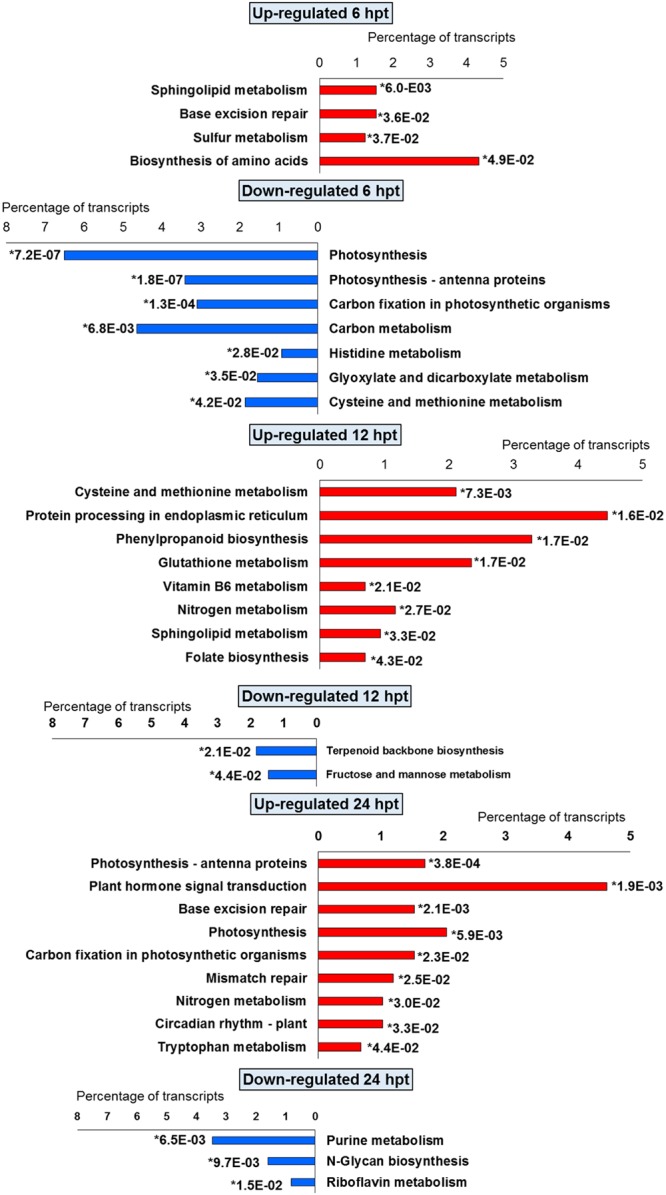
**Significantly (^∗^*p*-value ≤ 0.05) enriched pathways identified with KEGG Orthology-Based Annotation System (KOBAS) 2.0 in strawberry fruit following treatment with BTH at 6, 12, and 24 hpt**.

**FIGURE 5:**
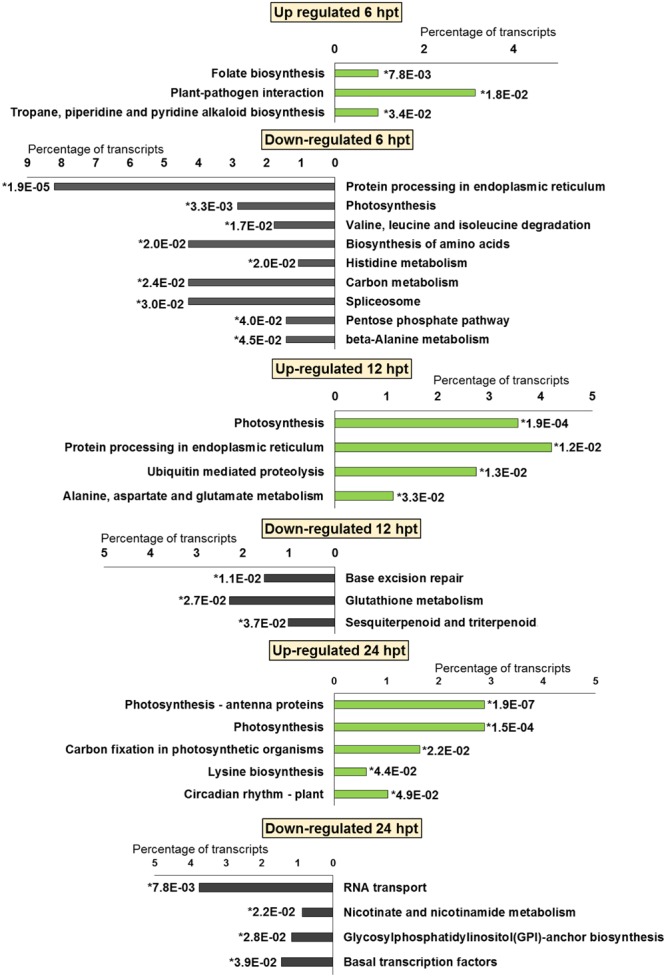
**Significantly (^∗^*p*-value ≤ 0.05) enriched pathways identified with KEGG Orthology-Based Annotation System (KOBAS) 2.0 in strawberry fruit following treatment with chitosan at 6, 12, and 24 hpt**.

**FIGURE 6:**
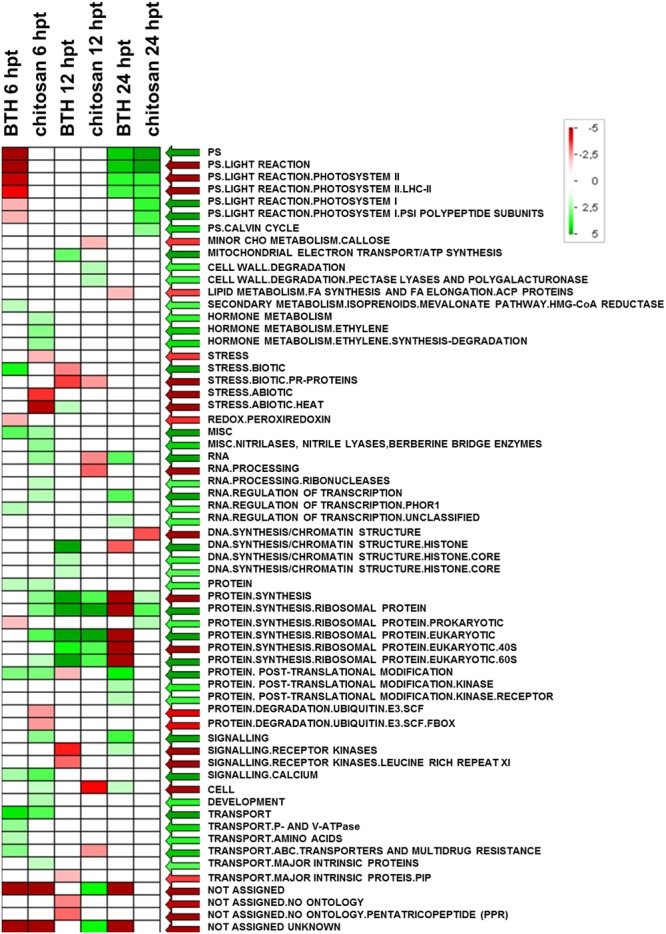
**Significantly enriched terms obtained using PAGEMAN software**. Green and red boxes indicate categories that are over-represented or under-represented, respectively, compared to control (for details, see also Materials and Methods).

The significant KEGG pathways were in agreement with the GO terms results, for both BTH and chitosan (**Figures 4** and **5**; Supplementary Table S4). The annotation of the functional classes was also performed according to the PAGEMAN functional bin classification (Thimm et al., 2004). The hierarchical tree structure of PAGEMAN allowed investigation the subcategories of genes involved in the elicitor treatment of the strawberry plants. These data are reported in **Figure 6** and Supplementary Table S5. The patterns primarily involved in the responses to the elicitors are discussed below.

### Light Phase of Photosynthesis

**FIGURE 7:**
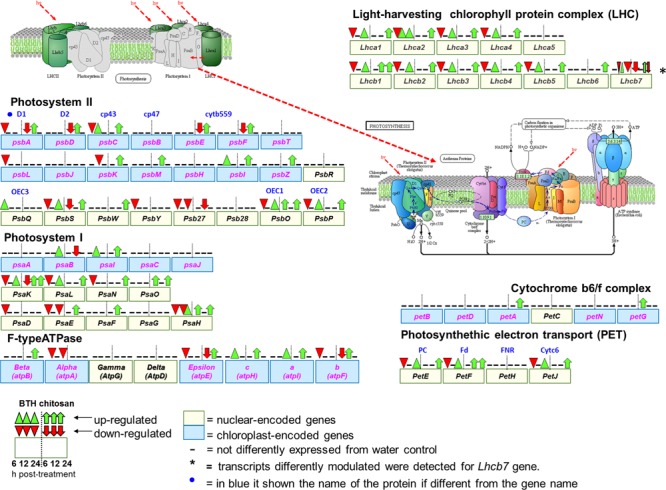
**Schematic representation of genes associated with photosynthesis (Kyoto Encyclopedia of Genes and Genomes, KEGG, fve 00195) and antenna protein (KEGG, fve00196) pathways in *Fragaria vesca* (http://www.ncbi.nlm.nih.gov/guide/genomes-maps/), as modulated by BTH and chitosan at 6, 12, and 24 hpt.** The original figure was modified to indicate the nuclear-encoded genes (yellow square), and chloroplast-encoded genes (blue square) (Rogalski et al., 2015). Cyt b559, cytochrome b559; OEC1 2, 3, oxygen-evolving complex1 2, 3; PC, plastocyanin; Fd, ferredoxin; FNR, ferredoxin-NADP^+^ reductase; Cytc6, cytochrome c6.

Comprehensive functional enrichment analysis showed that BTH and chitosan strongly affected the multi-step processes involved in the light phase of photosynthesis, when ATP and NADPH are produced (**Figure 7**). The overall analyses of the DEGs highlighted differences in the modulation of gene expression associated with the photosynthetic complex made up of both chloroplast-encoded genes and nuclear-encoded genes. All of the Light-Harvesting Chlorophyll (LHC) protein complex and the Photosynthetic Electron Transport (PET) proteins were nuclear-encoded, while all of the other photosystem components were encoded by both chloroplast and nuclear genes, such as photosystems I and II (PSI, PSII), the cytochrome b6/f complex (Cyb6/f) and F-type ATPase (**Figure 7**).

At 6 hpt, for BTH, several genes associated with the light phase of photosynthesis were down-regulated, which corresponded to 62.1% of the nuclear-encoded genes. At this time, chitosan down-regulated only 8.1% of the nuclear-encoded genes, which included *PsbS* (FC = -5.6), *Lhcb7* (FC = -3.2) and *PsaK* (FC = -2.4). In addition, at 6 hpt, both BTH and chitosan down-regulated 20% of the chloroplast-encoded genes (FC = -2 to -23.6). At this time, among the nuclear-encoded genes, BTH up-regulated only *PsbQ* (FC = 11.2), and chitosan *PsaH* and *PsaO* (FC = 2.4), while among the chloroplast-encoded genes, chitosan up-regulated only *petG* (FC = 11.8) (**Figure 7**; Supplementary Table S2).

At 12 hpt, a different trend was observed, as most of the previously down-regulated genes were restored or up-regulated. At this time, for BTH, 13.1% of the chloroplast-encoded genes were up-regulated (FC = 2.4 to 12.6), and only the *psbK* was down-regulated (FC = -2.1). While of nuclear-encoded genes only *Lhca2* (FC = -2.3), different transcripts of *Lhca7* (FC = 2.2 to 2.9 and -2.3), and *PsaH* (FC = -2.2) were affected. For chitosan at 12 hpt, there was up-regulation of 50% of the chloroplast-encoded genes (FC = 2.2 to 36.2), and of 16.6% of the nuclear-encoded genes (FC = 2.0 to 6.6). Only the *Psb27* was down-regulated (FC = -3.6).

At 24 hpt, there was up-regulation of nuclear-encoded genes, as 48.6% for BTH and 62% for chitosan, and these were involved primarily in the light phase photosynthetic complex. Only *Lhcb7* (FC = -2.8), *Psb27* (FC = -12.6), *PsaE* (FC = -2.1), and *atpA* (FC = -4.4) were down-regulated by BTH, as for only one transcript related to *Lhcb7* (FC = -2.5) by chitosan While of the chloroplast-encoded genes only the *psaB* (FC = 2.4) and *atpI* (FC = 3.1) were affected by BTH (**Figure 7**).

### Transcription Factors Associated with Light Signaling and Stress

**FIGURE 8:**
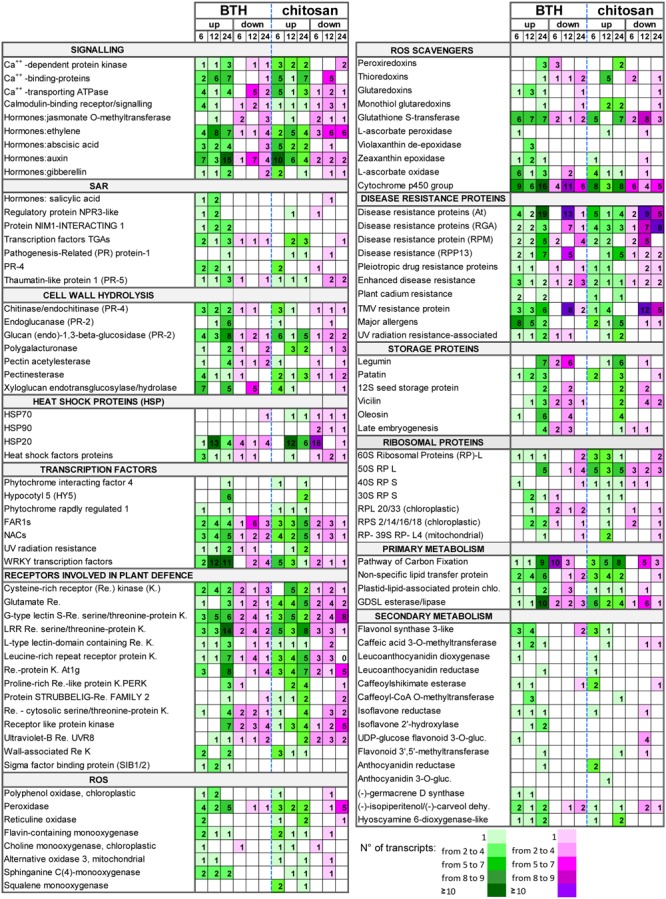
**Selection of DEGs (fold change; | FC | ≥ 2) up-regulated (up) and down-regulated (down) in strawberry fruit following treatment with BTH and chitosan at 6, 12, and 24 hpt.** The numbers of transcripts differently expressed are reported for each gene category. NPR3, NON-EXPRESSOR OF PATHOGENESIS-RELATED GENES; gluc, glucosyltransferase; dehy., dehydrogenase.

Among the TFs associated with light signaling, several *FAR1* genes were modulated. In particular, *FAR1-5* was strongly up-regulated by BTH and chitosan at 6 hpt (FC = 29.2 and 22.4, respectively) and at 24 hpt (FC = 13.2 and 6.1, respectively). Several *HYPOCOTYL 5* (*HY5*)-like genes were up-regulated by both elicitors at 24 hpt. In particular, *HY5 variant X2* was strongly up-regulated by BTH (FC = 26.8). The main TFs associated with stress responses were modulated by both elicitors, including the *NAC* genes. Among these, *NAC29* was strongly up-regulated by BTH and chitosan at 6 hpt (FC = 15.1 and 18.0, respectively) and at 24 hpt (FC = 7.0, for both elicitors) (**Figure 8**; Supplementary Table S2).

### ROS-Scavenger Metabolism

Among genes responsible for cell detoxification, the *PEROXIREDOXIN* (*PRX*), *THIOREDOXIN* (*TRX*), *GLUTAREDOXIN* (*GRX*), associated with ascorbate-independent thiol-mediated pathway, and the *GLUTATHIONE S-TRANSFERASE* (*GST*) genes involved in glutathione metabolism, were differently modulated by elicitors. In particular, the *PRX* genes *2-cys PRX, 1-cys PRX*, and *PRX-Q* were down-regulated at 6 hpt by BTH (FC = -2.3 to -8.6), and up-regulated at 24 hpt by both elicitors (FC = 2.6 to 8.1). The *TRX* genes were also affected by the BTH and chitosan treatments. In particular, at 6 hpt, both BTH and chitosan induced down-regulation of *TRX-M* (FC = -6.9 and -3.6, respectively) and, at 24 hpt, of *TRX-YLS8* (FC = -20.6 and -2.45, respectively), while at 12 hpt several *TRX* genes were up-regulated only by chitosan (FC = 2.8 to 17.8). In contrast, BTH primarily affected at 12 hpt the *GRX* genes, where the *GRX-C9*-like genes were up-regulated at all time points (FC = 2.3 to 3.6). For glutathione metabolism, *GST* was stimulated by the application of both elicitors. Using BTH, there was primarily up-regulation of the *GST* transcripts at 12 and 24 hpt (FC = 2.2 to 5.5). For chitosan, there was the greatest down-regulation of the *GST* transcripts at 12 hpt (FC = -2.2 to -22.4), with a prevalence of up-regulated transcripts at 24 hpt (FC = 2.1 to 33.0) (**Figure 8**; Supplementary Table S2).

### Systemic Acquired Resistance Signaling

Benzothiadiazole, but not chitosan, induced up-regulation of the *SALICYLIC-ACID-BINDING PROTEIN 2* genes at 6 hpt (FC = 6.4) and 12 hpt (FC = 21.4). The genes identified as *JASMONATE O-METHYLTRANSFERASE* were mainly down-regulated by both elicitors, with only one up-regulated at 12 hpt (Supplementary Table S2). For the genes associated with ET signaling, several transcripts were affected by BTH (FC = -7.5 to 18.6) and chitosan (FC = -4.9 to 9.8). However, up-regulated genes were prevalent at all time points in the fruit from the strawberry plants treated with BTH, with a different trend observed for chitosan, where down-regulated genes exceeded up-regulated genes.

Associated to SAR, the genes that encode PR proteins were affected by the elicitors. BTH up-regulated genes encoding PR4 at 6 and 12 hpt (FC = 2.0 to 11.4), while for chitosan this occurred only at 6 hpt (FC = 3.7 to 8.9). Although only one basic form of *PR1* was up-regulated by BTH treatment at 24 hpt (FC = 11.0), the *PR1* genes were up-regulated by chitosan at 12 and 24 hpt (FC = 4.4 to 21.4). The genes encoding *THAUMATIN* (PR5 function), *CHITINASE* and *ENDOCHITINASE* (PR4 function), and *GLUCAN 1,3-BETA-GLUCOSIDASE, ENDOGLUCANASE* and *GLUCAN ENDO-1,3-BETA-GLUCOSIDASE* (PR2 function) were mainly up-regulated by both elicitors at 6 and 24 hpt (**Figure 8**; Supplementary Table S2). The SAR-regulating proteins were mainly up-regulated by BTH. In particular, the *NON-EXPRESSOR OF PATHOGENESIS-RELATED GENES REGULATORY PROTEIN3* (*NPR3)-LIKE* was up-regulated at 6 and 12 hpt (FC = 2.2 to 4.4), and the genes *NIM1-INTERACTING* (*NIMIN*) 1 and 2 at all time points (FC = 2 to 18.8). Among the TFs involved in defense mechanisms, several *TGA*-3, -4, -5, and -6 TFs were mainly up-regulated at all time points by BTH (FC = -2.6 to 15.3), and at 12 and 24 hpt by chitosan (FC = -3.2 to 12.3). Several TF *WRKY* genes, such as *WRKY12, WRKY21, WRKY25, WRKY31, WRKY33, WRKY40, WRKY46, WRKY61*, and *WRKY70*, were mainly up-regulated by BTH at 12 and 24 hpt (FC = 2.1 to 13.0). In contrast, only a few of the *WRKY* genes, such as *WRKY20, WRKY50, WRKY51, WRKY53*, and *WRKY57*, were up-regulated by chitosan, especially at 6 hpt (FC = 2.1 to 4.6). *SIGMA FACTOR BINDING PROTEIN 2*, which is involved in plant defense, was up-regulated by BTH at all time points (FC = 2.1 to 9.1) (**Figure 2**; Supplementary Table S2).

### Disease-Resistance Proteins

In terms of disease resistance, more than 150 genes were stimulated by BTH and chitosan, which were related to the pleiotropic *DRUG RESISTANCE, DISEASE RESISTANCE RPPS, RGAS, RPMS, AGT, ENHANCED DISEASE RESISTANCE 2, PLANT CADMIUM RESISTANCE 2, UV RADIATION RESISTANCE*, and *TMV RESISTANCE*. Wide variability of gene expression was observed for both BTH (FC = -4.1 to 13.0) and chitosan (FC = -6.9 to 5.0), and only a few of these genes were affected at all of the time points.

The overall view for the disease resistance genes showed a greater number of up-regulated than down-regulated genes at 6 and 24 hpt for BTH, while for chitosan, at 24 hpt, the numbers of up-regulated and down-regulated genes were similar. For both elicitors, the down-regulated genes exceeded up-regulated genes at 12 hpt (**Figure 8**; Supplementary Table S2).

### Heat Shock Proteins

Among the different HSPs, the small HSPs (molecular weight, 12–40 kDa), indicated as *HSP20*, were the most affected by both BTH and chitosan, although *HSP70* and *HSP90* were also modulated. At 6 hpt, *HSP20* was down-regulated by chitosan (FC = -2.2 to -15.3), whereas at 12 hpt the up-regulated transcripts exceeded those down-regulated (FC = 2.2 to 38.8 and 2.2 to 11.5, for BTH and chitosan, respectively). A prevalence of up-regulated *HSP* genes was also observed at 24 hpt (**Figure 8**; Supplementary Table S2).

### Allergens

Benzothiadiazole and chitosan induced up-regulation of several allergen-related genes. In particular, the DEGs induced by both elicitors encoded the allergen homologs to *PRU AR1* from cherry, *PRU AV1* from apricot, *MAL D1* from apple, and the pollen allergens *OLE E10* from olive and *PHL L11* from grasses. BTH promoted up-regulation of these genes mainly at 6 and 12 hpt (FC = 2.1 to 9.1), while chitosan did the same mainly at 24 hpt (FC = -5.8 to 11.0) (**Figure 8**; Supplementary Table S2).

### Storage Proteins

There was strong induction of genes associated with storage proteins, with high RPKM in the treated fruit. Several genes, including *LEGUMIN A* and *B, PATATIN, 12S SEED STORAGE PROTEIN, LATE EMBRYOGENESIS, VICILIN* and *OLEOSIN*, were affected by both elicitors. After the moderate down-regulation seen at 12 hpt (maximum FC = -5), which was primarily for BTH, at 24 hpt, both elicitors showed their greatest up-regulation of these transcripts (maximum FC = 53.8 and 11.9, for BTH and chitosan, respectively) (**Figure 8**; Supplementary Table S2).

### A Model of Elicitor–Plant Signaling and Regulation in Strawberry Fruit

**FIGURE 9:**
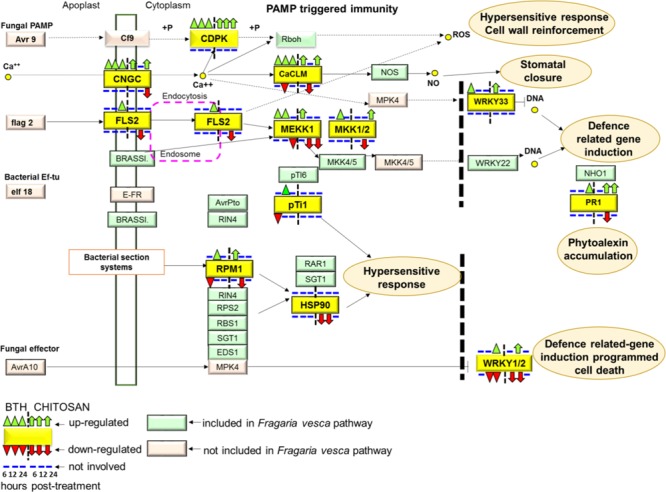
**The plant pathogen interaction pathway, Kyoto Encyclopedia of Genes and Genomes (KEGG): fve04626, in *Fragaria vesca* (http://www.ncbi.nlm.nih.gov/guide/genomes-maps/).** Up-regulation and down-regulation of the specific pathways elicited in strawberry fruit by BTH and chitosan at 6, 12 and 24 hpt. CNGC, cyclic nucleotide gated channel; CDPK, calcium-dependent protein kinase; CaCML, calcium-binding protein; WRKY, transcription factor 33; FLS2, LRR receptor-like serine/threonine-protein kinase FLS2; MEKK1, mitogen-activated protein kinase kinase 1; MKK1, mitogen-activated protein kinase kinase 1; pTi1, pto-interacting protein 1; RPM1, disease resistance protein RPM1; HSP90, heat shock protein 90; PR1, pathogenesis-related protein 1; WRKY1/2, WRKY transcription factor 1/2.

In terms of the Plant–Pathogen Interactions based on the KEGG pathway of *Fragaria vesca* (fve04626)^9^, BTH and chitosan showed differential modulation of the expression of genes that encoded 13 proteins included in this pathway (**Figure 9**; Supplementary Table S4). PAMP-triggered immunity constitutes the first line of inducible defense against infectious diseases. Several genes involved in this primary response linked to cytosolic Ca^2+^ concentrations, including cyclic nucleotide gated channel (*CNGC*), calcium-dependent protein kinase (*CDPK*), and calcium-binding protein CML (*CaMCML*) were mainly up-regulated at all time points by BTH and at 6 and 24 hpt by chitosan. Increase of Ca^2+^ are also regulator for production of ROS and localized programmed cell death/hypersensitive response (**Figure 9**). Otherwise, flagellin receptor FLS2, with the mitogen-activated protein kinases (MEKK1; MKK1/2) signaling pathway, were mainly activated by BTH. Associated to second layer of immunity termed ETI, the modulation on the time according both elicitors of genes encoded for disease-resistance proteins, represented by RPM1, as well as the down-regulation of HSP90 genes at 6 and 12 hpt were shown. Finally, elicitors-triggered transcriptome reprogramming showed a different modulation of WRKY33 and WRKY1/2 TFs and PR1 genes (**Figure 9**).

**FIGURE 10:**
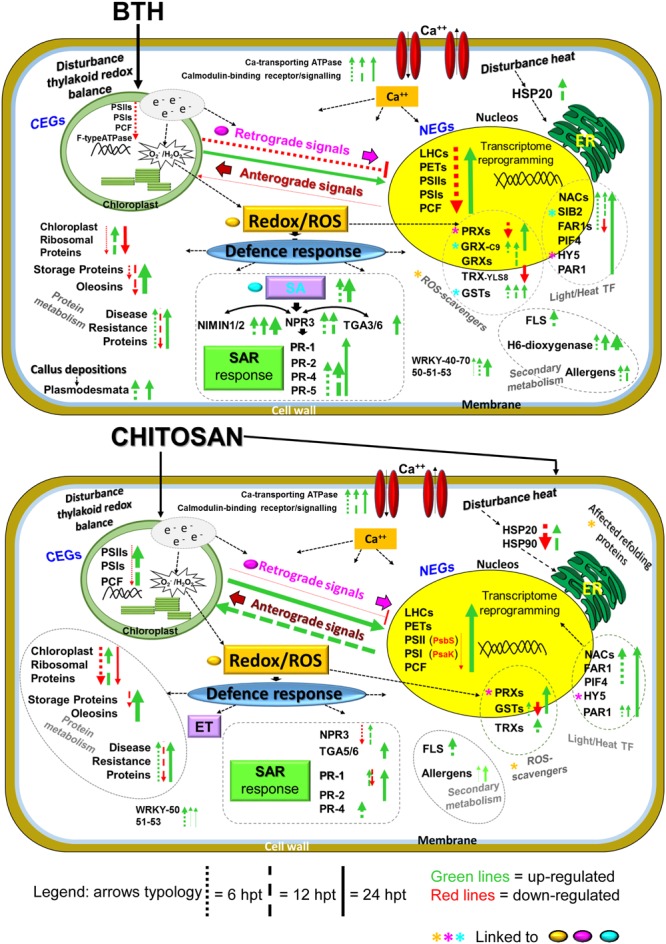
**Overview of the proposed transcriptome reprogramming model in strawberry fruit following preharvest treatment of the strawberry plants with BTH and chitosan at 6, 12, and 24 hpt, according to the major genes that were up-regulated and down-regulated.** The thickness of the arrows indicates the number of genes and the fold-change values compared to the water control. NEG, nuclear-encoded gene; CEG, chloroplast-encoded gene; LHC, light harvesting complex; PET, photosynthetic electron transport; PSI, photosystem I; PSII, photosystem II; PCF, photosynthetic carbon fixation; e^-^, electrons; ROS, reactive oxygen species; Ca^++^, calcium; SA, salicylic acid; ET, ethylene; NPR3, non-expressor of pathogenesis-related 3; NIMIN1, NIM1-interacting; TGA genes; PR1, PR4, PR2; PR5, pathogenesis related; SAR, systemic acquired resistance; HSP, heat shock protein; GRX, glutaredoxin; GST, glutathione-*S*-transferase; TRX, thioredoxin; PRX, peroxiredoxin; HY5, HYPOCOTIL-5; FLS, flavonol synthase; H6-dioxygenase, hyoscyamine 6-dioxygenase; PIF4, PHYTOCHROME INTERACTING FACTOR4; PAR1, PHYTOCHROME RAPIDLY REGULATED1 SIB2, SIGMA protein binding 2; WRKY, WRKY genes, TF, transcription factors. For further details, see main text, **Figure 8** and Supplementary Table S2.

A hypothetical model for the gene expression profiles in these strawberry fruits following application of the two elicitors to the plants, reported in **Figure 10**, will be discussed later.

### RT-qPCR Validation of the DEGs

**FIGURE 11:**
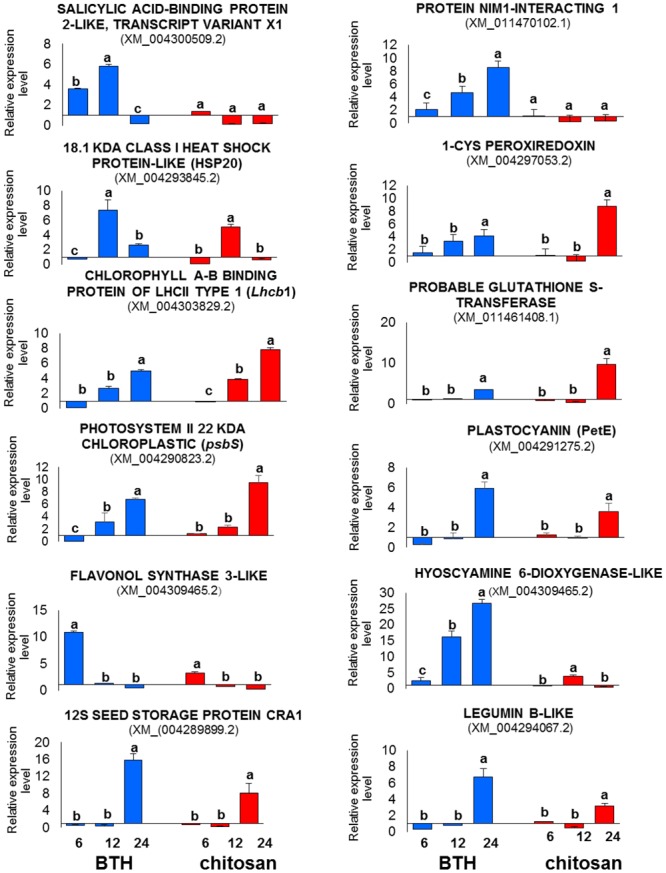
**RT-qPCR validation of 12 representative genes differentially expressed in the RNA-Seq analysis in strawberry after BTH and chitosan treatments at 6, 12, and 24 hpt.** Expression levels of each sample were normalized according to the *18S* and *ACTIN* reference genes validated according to qBase+ software in CFX Connect Real-Time PCR Detection System (Bio-Rad Laboratories) (coefficient of variation [CV] of normalized reference genes = 0.128; *M*-value reference gene expression stability = 0.3732 – Recommended stability value: CV < 0.25; *M* < 0.5). Relative expression values were determined against the average value of the water control sample. Each experimental replicate was determined as two technical replicates (*n* = 4). Data are means ± SD, and values with different letters are significantly different at *P* ≤ 0.05, according to Duncan’s multiple range tests.

The relative expression data provided by RT-qPCR were consistent with the profiles detected by RNA-Seq at all of the time points, with confirmation of the trends of up-regulation and down-regulation of all of the genes analyzed (**Figure 11**).

## Discussion

Benzothiadiazole and chitosan have been shown to reduce gray mold and *Rhizopus* rot following preharvest (Feliziani et al., 2015) and postharvest (Romanazzi et al., 2013) treatments, and to modulate selected defense genes (Landi et al., 2014). To contribute to the understanding of the whole transcriptional changes induced by BTH and chitosan in strawberry fruit, following preharvest application of these elicitors to the plants, RNA-Seq data were generated and validated by RT-qPCR. The differences in the magnitudes of changes observed between the RT-qPCR and RNA-Seq data relate to the differences between these approaches for gene expression analysis.

The RNA-Seq analysis carried out at 6, 12, and 24 hpt revealed that more than 5,000 genes were differentially expressed for each elicitor, as compared to the control. However, at each time, less than 21% of the transcripts were affected in the same way by both elicitors, and within each elicitor response, less than 1% of the transcripts were modulated at all of the times analyzed. This shows the great variability of such gene modulation over time. Enrichment analyses showed the involvement of genes associated with the light phase of photosynthesis, HSPs, storage proteins, and defense signaling. However, these BTH and chitosan treatments modulated the gene transcripts associated to photosynthesis in different ways according to time and, partially, to the chloroplast or nuclear localization of the genes (Rogalski et al., 2015).

Briefly, at 6 hpt, BTH induced significant down-regulation of several photosynthetic nuclear-encoded genes, including the *Lhc* genes. LHC has an important role in regulation and dissipation of excess energy flow under light stress. The expression of *Lhc* is coordinately repressed when the energy input through the antenna protein systems exceeds the requirement for CO_2_ assimilation (Külheim et al., 2002; Teramoto et al., 2002). Conversely, in the early phase after treatment with chitosan, there was down-regulation of few nuclear-encoded genes as *Lhcb7, Psak*, and *PsbS*. The *PsbS* gene encodes for a crucial pH-sensing protein in the non-photochemical quenching (NPQ) process (Ruban, 2016). NPQ is the thermal energy dissipation process induced by high light protonation that causes pH changes in the thylakoid. The function of NPQ is thus to prevent damage of the photosystems that control the generation of ^1^O_2_ (Roach and Krieger-Liszkay, 2014; Ruban, 2016). On the other hand, at 6 hpt, a few chloroplast-encoded genes were primarily down-regulated by both elicitors, including the *psbA* gene. *PsbA* encodes the D1 protein that with the D2 protein constitutes the core reaction of PSII, and has a role in protecting PSII, which is highly susceptible to photo damage, as a specific sensor of ROS (Huo et al., 2015). Although the NPQ activation depends on the PsbS protein, the pH change of the thylakoid is mainly generated by electron transport, which involves oxidation of water in the oxygen-evolving complex. In this regard, we note the association of BTH treatment with up-regulation of *PsbQ*, which codes for a protein that belongs to the oxygen-evolving complex in PSII. Previous studies have indicated that PsbQ is the target for effector-protein-triggered immunity in *Pseudomonas syringae* (Romero-Puertas et al., 2008), and is required for full deployment of ROS associated with plant defenses (Rodríguez-Herva et al., 2012). Therefore, despite the observed differences, both elicitors impact on ROS production.

At 12 hpt, a reversal of the trend occurred in the strawberry fruit. In particular, chitosan induced up-regulation of the main chloroplast genes, which were initially down-regulated, while at 24 hpt, the nuclear genes were mainly up-regulated by both elicitors. The expression of the photosynthetic genes changes according to the high or low input of light, with this being down-regulated or up-regulated, respectively (Häusler et al., 2014). Previous studies have frequently shown down-regulation of photosynthesis associated with pathogen attacks (Berger et al., 2007), environmental stress (Song et al., 2014; Kosová et al., 2015) and toxic effects of fungicides (Petit et al., 2012). We have shown that the resistance inducers tested here that are effective in disease control, also affect the photosynthetic processes without destroying them. Indeed, the systems were regenerated and an overexpression of the genes involved was observed at the later time, which appears to be induced to restore the equilibrium and for the increased protein metabolism that is useful in plant defense. Proteomic and biochemical analyses of resistant and susceptible plants have shown that the ability to maintain active photosynthesis during an infection is a crucial element in plant defense (Zhang et al., 2013). In this regard, the role of photosynthesis in non-photosynthetic fruit tissue and on its metabolism is not clear. For the strawberry fruits analyzed in the present study, green tissues were associated only with achenes (Meyerhoff and Pfündel, 2008). However, a study on tomato has indicated that ripe fruits are unlikely to be net assimilators of CO_2_, despite the high levels of expression of the photosynthetic genes, which suggests that these have a role in the improvement of fruit quality (Cocaliadis et al., 2014). The present data suggest that these elicitors influenced chloroplast functionality in different ways, with effects on the network transcriptome responses. The localization of the genes that encode the chloroplast proteins implies that there are molecular and physiological mechanisms that coordinate nuclear and plastid gene expression (Jarvis and López-Juez, 2013). This led to the development of the concept of retrograde signaling from chloroplast to nucleus (Koussevitzky et al., 2008; Kleine and Leister, 2013). However, the signaling between chloroplasts and the nucleus is bidirectional. In anterograde regulation, for adequate plastid development (Lefebvre-Legendre et al., 2015), nuclear-encoded regulators can modify the expression of both chloroplast and nuclear genes, even at the post-transcriptional level (Kleine and Leister, 2013). These signaling networks are dependent on plastid developmental and functional stage, intracellular distance, time scale, and target place of action (Szechyñska-Hebda and Karpiñski, 2013). In the present study, not all of the photosynthetic genes were affected by BTH and chitosan, and some of them showed different trends. This might be linked to the evolution of transduction signals that can be independent (e.g., *PsbS, Lhc*; Engelken et al., 2010), unique (e.g., *psbE, psbF*; Kiss et al., 2012), or associated with different protein characteristics (e.g., *Lhcb7*; Klimmek et al., 2006).

According to the data from the present study, the gene expression fluctuations of the affected chloroplast genes occurred from 6 to 12 hpt, while for the nuclear genes, this was mostly from 6 to 24 hpt, which stresses the importance of their intracellular localisation. Several candidates of retrograde signaling pathways have been proposed, including metabolite abundance, ROS pathways, and photosynthetic pigments (Singh et al., 2015). However, it has been recently suggested that all of these can be traced back to photosynthetic electron transfer energy balance/imbalance as the initiator of the chloroplast retrograde signaling cascade (Foyer et al., 2012; Gollan et al., 2015). Chloroplasts coordinate cellular activities and functions under stress responses, to promote survival against environmental perturbations (Gururani et al., 2015). This behavior can be used as a defense strategy, which affects carbohydrate metabolism in the stressed plant tissues. As an adaptive response to biotic and abiotic stresses, this will allow the plant to invest resources in the suddenly required defense responses, without this being debilitating to the plant. In this context, the present data highlight that the elicitors affect the chloroplasts through their actions on the electron flow, and consequently the redox/ROS status. This induces a transcriptome reprogramming in the nuclear genome, which includes up-regulation of defense-related genes (Driedonks et al., 2015; Gollan et al., 2015).

The production of specific ROS might be strictly involved in the elicitor responses linked to the chloroplast. However, up-regulation of the ROS scavenger was not observed in the early phases after these treatments. Whether ROS act by damaging the cell protective or signaling factors depends on the delicate equilibrium between ROS production and scavenging. Hence, ROS can damage cells as well as initiate responses, such as gene expression modulation and cellular immune responses (Gill et al., 2013). In terms of the role of ROS in the signaling networks, the *PRX* ROS-scavenger genes affected in the present study showed the same fluctuations in their expression that were observed for the photosynthetic nuclear-encoded genes, which suggests similar regulatory networks. PRX acts as an intracellular redox sensor and it transmits information related to the cellular levels of ROS (Awad et al., 2015). A role for PRX as a retrograde signal during oxidative stress under light conditions has also been suggested (Dietz, 2011).

Up-regulation of the *TRX* genes at 12 hpt with chitosan might have a role in the detoxification mechanisms (Hanschmann et al., 2013). The TRX detoxificant proteins are known to regulate numerous photosynthetic enzymes, and cross-talk between the plastid and the TRX system has been suggested to mediate light-dependent activation of primary photosynthetic reactions in plant chloroplasts, through reduction of disulphide bridges in redox-regulated enzymes (Meyer et al., 2012; Nikkanen et al., 2016). Based on the present data, we note that chitosan induced the simultaneous over-expression of chloroplast-encoded genes and *TRX*s at 12 hpt. However, other analytical approaches will be needed to clarify which molecules are actually involved in the signaling network.

At 12 hpt, KEGG analysis showed different involvement of the ‘glutathione metabolism pathway,’ which was here represented by the *GST* genes that were up-regulated by BTH and down-regulated by chitosan. GST is known as a cell detoxification system, and it is involved in the NPR1-independent SA-mediated pathway (Ghanta et al., 2011). In the same way, *GRX-C9* was induced only by BTH, and this is SA dependent (Herrera-Vásquez et al., 2015), in agreement with the SAR responses known to be induced by BTH (Görlach et al., 1996).

The present data suggest that the different initial impacts of these two elicitors on chloroplast functionality are pivotal keys that steer the cascade signaling pathways. We have highlighted here the predominant influence of BTH (an analog of SA) on strawberry genes linked to LHC components, and subsequently genes of other photosynthetic complexes that are mainly involved in the nuclear genes network. Previous studies have shown that the application of high concentrations of SA to *Arabidopsis* leaves rapidly induces stomatal closure and reduces the electron transport rate (Janda et al., 2012). Typically, high light-induced changes in stomatal conductance causes rapid repression of *LHC* genes and results in a photo-respiratory oxidative burst that helps to inform the cell of the redox changes in the PET chain, and to influence expression of photosynthetic genes (Foyer et al., 2012).

Strawberry plants treated with chitosan underwent partial photo-inhibition, as seen by down-regulation of specific genes in the strawberry fruit. The early responses to chitosan were down-regulation of genes encoding HSPs that are normally induced by abiotic and biotic stress. In particular, several genes encoding HSP20 proteins were affected, which prevent thermal aggregation of proteins (Park and Seo, 2015). This response might be associated with the properties of chitosan. In contrast to BTH, when chitosan is applied to plant tissues, it forms a physical barrier that results in decreased transpiration (Romanazzi et al., 2009), which affects the sensitivity of both heat stress and light signaling, as underlined by the results of the enrichment analysis. Heat stress commonly leads to inhibition of photosynthesis in higher plants (Song et al., 2014). However, the early down-regulation of the *HSP* genes might have a negative effect on protein refolding, and thus affect ROS production (Zeeshan et al., 2016). This is indicated as the ‘protein processing in endoplasmic reticulum’ pathway that is associated with *HSP* down-regulation following chitosan spraying. This trend changed at 12 hpt, when up-regulation of the *HSP* genes was observed with both elicitors, which highlights again the elicitor-induced modulation and recovery of gene responses. Therefore, the impact of the elicitors on the strawberry tissues was like an induction of stress, which, in the early phase from treatment for BTH, was linked with photosynthetic process, while, for chitosan, it was mostly linked with a heat response. Then, cross-talk between the heat response and the photosystem processes can be suggested (Kindgren et al., 2012; Song et al., 2014; Zhou et al., 2014). The present study showed up-regulation of specific TFs, including the *NAC* family, which is involved in heat stress and photosynthesis responses (Huang et al., 2015; Shao et al., 2015), associated to light responses, with FAR1 and HY5 (Bae and Choi, 2008; Kindgren et al., 2012), and involved in ROS homeostasis signaling (Wang and Wang, 2015). In addition, the *HY5* genes are associated with retrograde signaling (Kindgren et al., 2012). This suggests that these TFs function in the regulation of multiple stress tolerance through modulation of transcriptome reprogramming responses.

One of the earliest signaling events after recognition of a pathogen is seen for the ion fluxes across the plasma membrane, which includes influx of Ca^2+^ into the cytosol (Lecourieuxn et al., 2006). The present data showed the involvement of Ca^2+^ signaling with both elicitors. Differences among the elicitors were observed through the analysis of the activation of the hormone-signaling molecules that are associated with the inducible immune responses. The DEG analysis showed that SA signaling was up-regulated with BTH, while ET metabolism (synthesis/degradation) was modulated by chitosan, with both occurring in the early phase after their application. However, hormonal cross-talk involving the *WRKY* TFs appears to have a major role in the induced hormonal changes that modulate disease and resistance (Spoel and Dong, 2008; Huot et al., 2014). *WRKY50, WRKY51*, and *WRKY53* TFs are known repressors of JA signaling and are involved in hormonal cross-talk (Pieterse et al., 2012), and in the present study they were induced by both elicitors. Up-regulation of several transcripts of the *WRKY70* and *WRKY40* genes was associated exclusively with BTH treatment, and these might have pivotal roles in determining the balance between SA-dependent and JA-dependent defense pathways (Kazan and Lyons, 2014).

Salicylic acid has been indicated as the principal signal in SAR (Fu and Dong, 2013; Coqueiro et al., 2015). SAR activation results in the coordinated production of PR proteins (Pieterse et al., 2012). Here, the GO analysis at 6 hpt indicated that the cell-wall and extracellular-matrix terms, and the xyloglucan transferase activity, were induced by BTH. This suggests that remodeling of the cell-wall architecture is important to enhance disease resistance, as well as the involvement of structural proteins with PR-protein functions.

PR1 is a useful molecular marker for the SAR response (Fu and Dong, 2013). BTH is known to mimic the defense-associated effects of SA (Görlach et al., 1996), and for this reason, up-regulation of the *PR1* gene was expected. Nevertheless, *PR1* was up-regulated by BTH only at 24 hpt. Other central regulators in the SAR response were up-regulated by BTH, including *NPR3* (a paralog of *NPR1*; Fu et al., 2012; Kuai et al., 2015) and the NIMIN proteins that are active at high SA concentrations (Fu and Dong, 2013) as regulators of late SAR genes (e.g., PR1), for prevention of their premature activation (Glocova et al., 2005; Weigel et al., 2005; Hermann et al., 2013). This is in agreement with our previous studies in strawberry fruit that showed that BTH did not affect *PR1* gene expression (Landi et al., 2014). The NIMIN–*NPR* connection might constitute a molecular device to monitor SA levels in diseased plants, which would allow the plant to translate gradually to an increasing gradient of the defense hormone SA in two clear decision steps, as early and late SAR gene expression. These mechanisms of regulation of SAR were not activated by chitosan. However, chitosan caused the induction of genes associated to PR proteins including *PR1*.

Starting from 12 hpt, the photosystem process recovered, with up-regulation of photosynthetic genes induced by both elicitors. This had strong consequences on protein metabolism. Indeed, the elicitors affected the protein network, which included ribosomal and storage proteins, as well as disease-resistance proteins. Plant adjustments to an altered environment require high numbers of novel proteins to be synthesized, as well as for proteins to be degraded (Kosová et al., 2015). The elicitors strongly affected modulation of the ribosomal genes, which were mainly up-regulated at 12 hpt and down-regulated at 6 and 24 hpt. The present results suggest that ribosomal proteins change in a stress-specific manner, as part of the adaptation to elicitor stress, which will probably have biased protein translation (Wang et al., 2013). Several genes that encoded resistance proteins were induced by the elicitors, and up-regulated genes were predominant at 6 hpt and again at 24 hpt, which underlines the modulation of defense-response genes according to time.

Massive induction was recorded for genes that encode storage proteins (e.g., legumins, 12S storage proteins, vicilins, patatins) and genes involved in lipid metabolism (e.g., oleosins). In particular, strong up-regulation was observed at 24 hpt for both elicitors. This suggests that the expression of genes that encode storage proteins correlates with over-expression of genes involved in the photosynthetic process. In addition to having essential roles for plant survival, the storage proteins have roles in defense mechanisms (Cândido et al., 2011) through their insecticidal and antimicrobial properties, as has been observed for vicilin and patatin (Banerji and Flieger, 2004).

## Conclusion

Our data confirm that plant responses to elicitors are dynamic processes that induce deep changes in the kind, quantity and timing of the genes involved. This establishes novel homeostasis between plants and their environment that can enhance plant defense mechanisms against pathogens. The crucial impact of BTH and chitosan on the photosynthetic process generally begins with down-regulation, followed by over-expression of fundamental photosynthetic genes. This helps to maintain the imbalance/balance of ROS/redox signaling, and attributes a key role to the chloroplasts as the sensors of environmental changes, which allows them to protect the photosynthetic apparatus from stresses. However, the specific characteristics of these resistance inducers, such as the formation a protective film on plant tissues by chitosan and the analogous actions of BTH compared to SA, drive the response network in the early phase after their application. The typical SA signaling during plant immunity was found to be associated with BTH. However, the involvement of PR proteins with both elicitors, and in particular of PR1, which is one of the SAR response markers, suggests SAR induction also for chitosan.

We have here highlighted that the resistance inducers BTH and chitosan that are effective in the control of postharvest diseases of strawberry (Feliziani et al., 2015) deeply modulate the cellular metabolism. The genes identified in the present study can represent markers to better elucidate plant/pathogen/resistance-inducer interactions and to design novel sustainable disease-management strategies.

## Author Contributions

LL performed the experiments, analyzed the data, and wrote the article; RMDMA performed most of the experiments, analyzed the data, and supervised and complemented the writing; SP supervised the writing; EF performed part of the experiments; FF designed the experiments, and supervised and complemented the writing; GR designed the experiments, supervised and complemented the writing, and coordinated the collaboration of the authors.

## Conflict of Interest Statement

The authors declare that the research was conducted in the absence of any commercial or financial relationships that could be construed as a potential conflict of interest.

The reviewer EA and handling Editor declared their shared affiliation, and the handling Editor states that the process nevertheless met the standards of a fair and objective review.

## Footnotes

1 https://usegalaxy.org/

2 ftp://ftp.ncbi.nlm.nih.gov/genomes

3 http://www.blast2go.com/b2glaunch

4 http://www.geneontology.org

5 http://www.genome.jp/kegg

6 http://mapman.gabipd.org/

7 http://biotools.umassmed.edu/bioapps/primer3_www.cgi

8 ftp://ftp.bioinfo.wsu.edu/species/Fragaria_x_ananassa/

9 http://www.ncbi.nlm.nih.gov/guide/genomes-maps/
