# Supplementary material for: Global Transcriptome Analysis and Identification of Differentially Expressed Genes in Strawberry after Preharvest Application of Benzothiadiazole and Chitosan
Source: Front Plant Sci. 2017 Feb 24;8:235. doi: 10.3389/fpls.2017.00235 (PMC5323413; doi:10.3389/fpls.2017.00235)
Supplement: Supplementary file 1 [file Table_1.docx]

| **Gene** | **transcript_ID** | **Primes sequence**  **(5’-3’)** | **TM (°C)** | **Amplicon size (bp)** | **PCR efficiency (%)** | **R^2^ of standard curve** |
| --- | --- | --- | --- | --- | --- | --- |
| ***18.1 kDa class I heat shock protein-like*** | XM_004293845.2 | F- ggaaggaaacctcgaactcc  R- acgcattgaagccttgatct | 63.8  63.7 | 245 | 99.4 | 0.990 |
| ***flavonol synthase 3-like*** | XM_004300274.2 | F- tcctagttccacggttccac  R- gcatatgcagaaggcacaga | 63.9  64.0 | 173 | 96.3 | 0.995 |
| ***hyoscyamine 6-dioxygenase-like*** | XM_004309465.2 | F-ggttgcctgagagtgagagg  R-tccctgaaagagggaggatt | 64.1  63.8 | 184 | 91.8 | 0.990 |
| ***1-Cys peroxiredoxin*** | XM_004297053.2 | F-ccctgacaagaaggtggtgt  R-cctttctgggaaggaaggtc | 64.0  63.8 | 234 | 99.6 | 0.993 |
| ***probable glutathione S-transferase*** | XM_011461408.1 | F- catggcctagtcccttttgt  R- taaggatcctcggaaagcaa | 62.8  63.5 | 238 | 97.3 | 0.989 |
| ***protein NIM1-INTERACTING 1*** | XM_011470102.1 | F- caaggcaggtgtgtgagatg  R- ttgaattttcgaaggacatgc | 64.5  63.8 | 191 | 98.4 | 0.993 |
| ***salicylic acid-binding protein 2-like, transcript variant X1*** | XM_004300509.2 | F- ggtctctgccaccagaagag  R- aaagatcattgcggatggag | 64.1  63.9 | 245 | 96.8 | 0.995 |
| ***chlorophyll a-b binding protein of LHCII type 1-like (Lhcb1)*** | XM_004303829.2 | F- gctgtgaagcttggttcctc  R- gcaaaggtctcaggatcagc | 64.0  64.0 | 233 | 97.6 | 0.991 |
| ***photosystem II 22 kDa protein, chloroplastic (PsbS)*** | XM_004290823.2 | F-gtgaccgtggtcagtttgtg  R-gctagagctcccttccctgt | 64.3  62.7 | 211 | 98.7 | 0.990 |
| ***plastocyanin (PetE)*** | XM_004291275.2 | F-tttggccattgagatccttc  R-taggagatcctcctcgctca | 62.2  63.4 | 196 | 99.3 | 0.996 |
| ***12S seed storage protein CRA1-like*** | XM_004289899.2 | F- gatccggagacaacaaggaa  R- gcgtccctgtaccctaacaa | 64.0  63.8 | 175 | 99.1 | 0.961 |
| ***legumin B-like*** | XM_004294067.2 | F- aaaacccaaggcgattcta  R- gccggagaagatgttgttgt | 64.0  63.8 | 156 | 94.8 | 0.957 |
| ***ACTIN ^a^*** | AB116565.1 ^b^ | F-cgaggctcaatccaaaagag  R-ggggcctcagttaggagaac | 59.95  60.07 | 159 | 97.3 | 0.994 |
| ***18S ribosomal RNA gene ^a^*** | AF163494.1 ^b^ | F-cgccaaggaacttgaatgaa  R-ttgcgttcaaagactcgatg | 61.14  59.99 | 198 | 102.7 | 0.996 |

**Table S1**: Primers selected for the RT-qPCR analysis. The qPCR eﬃciency of each primer pair determined using standard curves generated according of three triplicated cDNA pool dilutions (undiluted, 0.10, 0.01), was reported. PCR ampliﬁcation eﬃciencies calculated according to standard curves and regression coeﬃcients for the standard curves are reported for each primer pair.

**NOTE:** ^a^ = Reference genes (Landi *et al.*, 2014); ^b^ = National Center for Biotechnology Information (NCBI), accession number. **Abbreviations:** F = forward; R = reverse.
