# Supplementary material for: Global Transcriptome Analysis and Identification of Differentially Expressed Genes in Strawberry after Preharvest Application of Benzothiadiazole and Chitosan
Source: Front Plant Sci. 2017 Feb 24;8:235. doi: 10.3389/fpls.2017.00235 (PMC5323413; doi:10.3389/fpls.2017.00235)
Supplement: Supplementary file 3 [file Table_3.docx]

**Table S3:** Differentially expressed genes (DEGs), included in the significantly enriched Gene Ontology (GO) terms (see also **Table S2**).

| **GO-ID** | **Term** | **Category*** | **FDR** | **P-Value** | **N° annotated transcripts** | | **DEG sequences** |
| --- | --- | --- | --- | --- | --- | --- | --- |
|  |  |  |  |  | **DEGs set** | **Reference Transcriptome** |  |
| **BTH 6 hpt up-regulated DEGs (Total sequences in DEGs set: 668; in Reference Transcriptome: 13958)** | | | | | | | |
| GO:0010411 | xyloglucan metabolic process | P | 7.92E-03 | 1.95E-05 | 6 | 8 | XM_004303812.2, XM_004303811.2, XM_004293438.2, XM_004303813.2, XM_004293320.2, XM_004297592.2. |
| GO:0016762 | xyloglucan:xyloglucosyl transferase activity | F | 9.69E-04 | 1.04E-06 | 7 | 7 | XM_004303812.2, XM_004303811.2, XM_004293438.2, XM_004303813.2, XM_004293320.2, XM_004299154.2, XM_004297592.2. |
| GO:0005618 | cell wall | C | 1.77E-04 | 8.13E-08 | 26 | 147 | XM_004294698.2, XM_004291631.2, XM_011466217.1, XM_004303812.2, XM_004289992.2, XM_004299154.2, XM_011466298.1, XM_004298613.2, XM_004301276.2, XM_011469744.1, XM_004299606.2, XM_004297592.2, XM_004290441.2, XM_004288226.2, XM_004307769.2, XM_004293583.2, XM_004298614.2, XM_004287582.2, XM_004293438.2, XM_004293320.2, XM_004299670.2, XM_004293256.2, XM_004306891.2, XM_004290762.2, XM_004303811.2, XM_004303813.2. |
| GO:0005576 | extracellular region | C | 2.85E-02 | 7.43E-05 | 23 | 184 | XM_004294698.2, XM_004288226.2, XM_004291631.2, XM_004303812.2, XM_004288054.2, XM_004293583.2, XM_004298614.2, XM_011468274.1, XM_011462782.1, XM_004287582.2, XM_004293438.2, XM_004307238.2, XM_011471397.1, XM_004293320.2, XM_004289992.2, XM_004300379.2, XM_004299154.2, XM_004302147.2,, XM_004303174.2, XM_004292053.2, XM_004303811.2, XM_004303813.2, XM_004297592.2. |
| GO:0031012 | extracellular matrix | C | 3.9E-02 | 9.49E-05 | 3 | 0 | XM_004297630.2, XM_011468274.1, XM_004287582.2. |
| **BTH 6 hpt down-regulated DEGs (Total sequences in DEGs set: 424, in Reference Transcriptome: 14202)** | | | | | | | |
| GO:0009765 | photosynthesis, light harvesting | P | 3.08E-08 | 3.30E-11 | 11 | 14 | XM_004300624.2, XM_004310031.2, XM_004302588.2, XM_004307347.2, XM_004303865.2, XM_004303830.2, XM_004303829.2, XM_004294441.2, XM_011461859.1, XM_004293412.2, XM_004293531.2. |
| GO:0010207 | photosystem II assembly | P | 7.54E-03 | 2.78E-05 | 10 | 59 | XM_004287164.2, XM_004306562.2, XM_004309958.2, psbL_, XM_004304075.2, XM_004290823.2, XM_004306946.2, XM_004289663.2, XM_011469969.1, XM_004300527.2 |
| GO:0016168 | chlorophyll binding | F | 6.02E-03 | 2.12E-05 | 5 | 8 | XM_004310031.2, XM_004290823.2, XM_004304664.2, psbC_, XM_004293412.2. |
| GO:0009535 | chloroplast thylakoid membrane | C | 5.92E-08 | 8.17E-11 | 22 | 117 | XM_004287164.2, XM_004306562.2, XM_004302848.2, XM_004290823.2, XM_004289974.2, XM_004300527.2, XM_004296848.2, XM_004303852.2, XM_004310031.2, XM_004309958.2, psbL_, XM_004287562.2, atpA_, XM_004304664.2, atpE_, psbC_, atpF_, XM_004293412.2, psbA_, XM_004288072.2, XM_004291275.2, XM_011462623.1. |
| GO:0009523 | photosystem II | C | 3.51E-05 | 8.62E-08 | 10 | 28 | XM_004310031.2, XM_004309958.2, psbL_, XM_004287562.2, psbC_, XM_004293412.2, XM_004300527.2, psbA_, XM_004303427.2, XM_004303854.2. |
| GO:0009538 | photosystem I reaction center | C | 1.17E-03 | 3.40E-06 | 4 | 1 | XM_004302848.2, XM_004289974.2, XM_004302108.2, XM_004296848.2 |
| **BTH 12 hpt down-regulated DEGs (Total sequences in DEGs set: 536, in Reference Transcriptome: 14090)** | | | | | | | |
| GO:0043531 | ADP binding | F | 1.92E-04 | 2.95E-08 | 25 | 165 | XM_004296110.2, XM_004308648.1, XM_011463618.1, XM_011466157.1, XM_011471677.1, XM_011471501.1, XM_004309293.2, XM_011463356.1, XM_011463616.1, XM_011462413.1, XM_011471761.1, XM_004309391.2, XM_011463922.1, XM_004308942.2, XM_011470277.1, XM_004305104.2, XM_011460429.1, XM_011472123.1, XM_011467321.1, XM_011462862.1, XM_011464795.1, XM_004288027.2, XM_011470873.1, XM_011461418.1, XM_011470807.1. |
| GO:0045735 | nutrient reservoir activity | F | 4.91E-03 | 3.70E-06 | 8 | 19 | XM_011459700.1, XM_004289896.2, XM_004289898.2, XM_011459704.1, XM_011459702.1, XM_004294067.2, XM_004309837.2, XM_011459701.1. |
| GO:0016762 | xyloglucan:xyloglucosyl transferase activity | F | 4.02E-02 | 9.87E-05 | 5 | 9 | XM_004293322.2, XM_004293438.2, XM_004303813.2, XM_004293320.2\|, XM_004299154.2 |
| GO:0012511 | monolayer-surrounded lipid storage body | C | 1.83E-02 | 2.52E-05 | 4 | 2 | XM_004307729.2, XM_004292787.2, XM_004304601.2, XM_004292786.1. |
| **BTH 24 hpt up-regulated DEGs (Total sequences in DEGs set: 1185, in Reference Transcriptome: 13441)** | | | | | | | |
| GO:0009765 | photosynthesis, light harvesting | P | 8.60E-04 | 1.45E-06 | 11 | 14 | XM_004300624.2, XM_004310031.2, XM_004302588.2, XM_004303830.2, XM_004303829.2, XM_004303865.2, XM_004294219.2, psbE_, XM_004299300.2, XM_004294441.2, XM_004293412.2. |
| GO:0052543 | callose deposition in cell wall | P | 3.11E-02 | 2.01E-04 | 4 | 1 | XM_004309931.2, XM_004298544.2, XM_004299535.2, XM_004297619.2. |
| GO:0045735 | nutrient reservoir activity | F | 1.70E-03 | 3.65E-06 | 11 | 16 | XM_011459700.1, XM_004289898.2, XM_004289896.2, XM_004293660.2, XM_011459704.1, XM_004292530.2, XM_011459702.1, XM_004289897.2, XM_004294067.2, XM_004309837.2, XM_011459701.1. |
| GO:0016168 | chlorophyll binding | F | 6.03E-03 | 2.50E-05 | 7 | 8 | XM_004310031.2, XM_004290823.2, XM_004294219.2, XM_004304664.2, XM_004299300.2, psaB, XM_004293412.2. |
| GO:0012511 | monolayer-surrounded lipid storage body | C | 4.93E-03 | 1.94E-05 | 5 | 6 | XM_004307729.2, XM_004292787.2, XM_004299193.2, XM_004304601.2, XM_004292786.1. |
| GO:0009522 | photosystem I | C | 1.71E-02 | 9.43E-05 | 8 | 12 | XM_004306562.2, XM_004310031.2, XM_004302848.2, XM_004294219.2, XM_004304664.2, psaB, XM_004293412.2, XM_004302108.2. |
| **BTH 24 hpt down-regulated DEGs (Total sequences in DEGs set: 619, in Reference Transcriptome: 14003)** | | | | | | | |
| GO:0044444 | cytoplasmic part | C | 3.55E-02 | 5.45E-06 | 74 | 2718 | XM_011464018.1, XM_011472763.1, XM_011464425.1, XM_011466053.1, XM_004297452.2, XM_011464296.1, XM_011463992.1, XM_011460608.1, XM_004296600.2, XM_011462278.1, XM_004307142.2, XM_011469405.1, XM_004297249.2, XM_004294443.2, XM_011466865.1, XM_011472529.1, XM_004292567.2, XM_011471246.1, XM_011463481.1, XM_004293833.2, XM_004306279.2, XM_011467852.1, XM_004309495.2, XM_011461297.1, XM_011471049.1, XM_011472755.1, XM_004305501.2, XM_011471030.1, XM_011467934.1, XM_004303062.2, XM_004299135.2, XM_011466216.1, XM_011464191.1, XM_004288296.2, XM_011470507.1, XM_011464543.1, XM_004306540.2, XM_011472765.1, XM_011471009.1, XM_004309488.2, XM_004305419.1, ndhJ, XM_011464359.1, XM_011472559.1, XM_011460348.1, XM_004288614.2, XM_011462164.1, XM_004297549.2, XM_004305674.2, XM_011459116.1, XM_004299596.2, XM_011460601.1, XM_011466652.1, XM_004290031.2, XM_011467762.1, XM_011471103.1, XM_011465991.1, XM_004291798.2, XM_004305593.2, XM_011465452.1, XM_004306508.2, XM_011466526.1, XM_011462834.1, XM_004308557.2, XM_011462565.1, XM_011466701.1, XM_004295473.2, XM_011462857.1, XM_004287161.2, XM_004287164.2, XM_004309939.2, XM_004297676.2, XM_004288000.2, ycf3. |
| **Chitosan 6 hpt down-regulated DEGs (Total sequences in DEGs set: 407, in Reference Transcriptome: 14219)** | | | | | | | |
| GO:0042542 | response to hydrogen peroxide | P | 2.47E-02 | 9.89E-06 | 9 | 42 | XM_004303435.2, XM_004308002.2, XM_004308003.2, XM_004287097.2, XM_011460576.1, XM_011462562.1, XM_004297316.2, XM_011462181.1, XM_004287481.2. |
| GO:0009644 | response to high light intensity | P | 2.47E-02 | 1.14E-05 | 10 | 55 | XM_011466635.1, XM_004303435.2, XM_004308002.2, XM_004308003.2, XM_004287097.2, XM_011460576.1, XM_011462562.1, XM_004297316.2, XM_011462181.1, XM_004287481.2. |
| GO:0009408 | response to heat | P | 4.21E-02 | 2.58E-05 | 12 | 90 | XM_004303435.2, XM_004307875.2, XM_004308003.2, XM_004287097.2, XM_011462562.1, XM_004299261.2, XM_004297316.2, XM_011462181.1, XM_011465521.1, XM_004303684.2, XM_004291003.2, XM_004291004.2. |
| **Chitosan 12 hpt up-regulated DEGs (Total sequences in DEGs set: 889, in Reference Transcriptome: 13737)** | | | | | | | |
| GO:0015979 | photosynthesis | P | 1.44E-02 | 2.22E-05 | 29 | 177 | XM_004309610.2, XM_011470973.1, XM_004302245.2, XM_011462278.1, XM_011463408.1, psbK, XM_011463482.1, XM_004300101.2, psbF, psaI, psbM, psbZ, XM_004309782.2, petA, XM_004293677.2, psbD, XM_011462199.1, atpH, psbE, XM_004289663.2, psbC, XM_004303830.2, XM_004289974.2, XM_004303427.2, ycf4, XM_004304664.2, XM_011469969.1, XM_004287562.2, psbA. |
| GO:0031625 | ubiquitin protein ligase binding | F | 3.41E-02 | 9.59E-00 | 6 | 13 | XM_011466957.1, XM_011466959.1, XM_011466956.1, XM_011466955.1, XM_011466954.1, XM_011466958.1. |
| GO:0009523 | photosystem II | C | 6.02E-04 | 1.85E-07 | 13 | 25 | XM_004309610.2, XM_011470973.1, XM_004302245.2, psbK, psbF, psbM, psbZ, psbD, psbE, psbC, XM_004303427.2, XM_004287562.2, psbA. |
| GO:0009535 | chloroplast thylakoid membrane | C | 2.89E-03 | 3.10E-06 | 24 | 115 | XM_004309610.2, XM_004290836.2, psbK, XM_004299350.2, XM_011463482.1, XM_004291322.2, XM_011459814.1, psbF, psaI, psbM, psbZ, petA, XM_004293677.2, psbD, atpH, atpI, psbE, psbC, XM_004289974.2, ycf4, XM_004304664.2, atpB, XM_004287562.2, atpF, psbA. |
| **Chitosan 12 hpt down-regulated DEGs (Total sequences in DEGs set: 779, in Reference Transcriptome: 13847)** | | | | | | | |
| GO:0043531 | ADP binding | F | 4.44E-02 | 1.02E-04 | 24 | 166 | XM_011463913.1, XM_011467315.1, XM_011463356.1, XM_004296111.2, XM_004297284.2, XM_011470873.1, XM_011472504.1, XM_011470681.1, XM_004301401.2, XM_004300993.2, XM_004301430.2, XM_004301305.2, XM_011464795.1, XM_004306324.1, XM_011471502.1, XM_004296105.2, XM_011470277.1, XM_011466787.1, XM_011463922.1, XM_011471399.1, XM_011463616.1, XM_011462413.1, XM_011462862.1, XM_004309293.2. |
| GO:0005488 | binding | F | 4.28E-02 | 9.19E-05 | 499 | 8230 | XM_011465722.1, XM_011461701.1, XM_004289152.1, XM_004307734.2, XM_004300694.2, XM_004291631.2, XM_011459427.1, XM_004302796.2, XM_004294973.2, XM_011462527.1, XM_004293478.2, XM_004300460.2, XM_011467987.1, XM_011471850.1, XM_004308399.2, XM_011466032.1, XM_004302147.2, XM_011464683.1, XM_004299434.2, XM_004288273.2, XM_011466797.1, XM_011471462.1, XM_004291013.2, XM_004287576.2, XM_011467342.1, XM_011468975.1, XM_011463999.1, XM_011472573.1, XM_011465826.1, XM_011469993.1, XM_011459761.1, XM_004299704.2, XM_004293431.2, XM_011465752.1, XM_004290717.2, XM_011461068.1, XM_004299566.2, XM_004294761.2, XM_004289367.2, XM_011463913.1, XM_011470023.1, XM_011465646.1, XM_004287120.2, XM_004301562.2, XM_011467315.1, XM_004305282.1, XM_011471309.1, XM_011468666.1, XM_011471894.1, XM_011463866.1, XM_004289971.2, XM_011466563.1, XM_004289204.2, XM_011466999.1, XM_011468112.1, XM_011469872.1, XM_011468627.1, XM_011459955.1, XM_011461630.1, XM_011471001.1, XM_011468534.1, XM_011466042.1, XM_004300457.2, XM_011468188.1, XM_004295210.2, XM_011467844.1, XM_011464920.1, XM_011472658.1, XM_011462782.1, XM_004297037.2, XM_004288272.2, XM_004297441.2, XM_004308148.2, XM_011465033.1, XM_004287122.2, XM_011468359.1, XM_011466901.1, XM_004302733.2, XM_011469405.1, XM_011463356.1, XM_011467046.1, XM_011461276.1, XM_011471253.1, XM_011465043.1, XM_004296766.2, XM_011464777.1, XM_011465577.1, XM_004289669.2, XM_004287223.2, XM_004295442.2, XM_004295888.2, XM_011468083.1, XM_011460239.1, XM_011459748.1, XM_011469658.1, XM_004292971.2, XM_004302063.2, XM_011466920.1, XM_004310072.2, XM_011472284.1, XM_011461148.1, XM_004296111.2, XM_011464341.1, XM_004289778.2, XM_004297251.2, XM_011459195.1, XM_004296442.2, NM_001280066.1, XM_011461192.1, XM_011461408.1, XM_011469587.1, XM_011472529.1, XM_004306804.2, XM_011460911.1, XM_004310009.2, XM_011466559.1, XM_011461730.1, XM_011471744.1, XM_011471375.1, XM_011462267.1, XM_011461195.1, XM_011469017.1, XM_004289394.2, XM_011467749.1, XM_004302417.2, XM_011459605.1, XM_004297284.2, XM_011460882.1, XM_011466793.1, XM_011463987.1, XM_011471246.1, XM_011470873.1, XM_004297629.2, XM_004290121.2, XM_011471386.1, XM_011471326.1, XM_011469795.1, XM_011460859.1, XM_011460525.1, XM_011467852.1, XM_004309495.2, XM_004290452.2, XM_011460922.1, XM_004287769.2, XM_004288088.2, XM_004296338.2, XM_011464187.1, XM_011468496.1, XM_004289214.1, XM_004309436.2, XM_011467261.1, XM_004299721.2, XM_011469241.1, XM_004289967.2, XM_004289487.2, XM_011466767.1, XM_011464093.1, XM_011472504.1, XM_004306286.2, XM_004288435.2, XM_011471874.1, XM_004300442.2, XM_011464059.1, XM_011463262.1, XM_004289189.2, XM_011470681.1, XM_011469157.1, XM_011467589.1, XM_004301098.2, XM_011462350.1, XM_011472531.1, XM_004302807.2, XM_004293042.2, XM_011460716.1, XM_011464058.1, XM_004287366.2, XM_004305501.2, XM_011468371.1, XM_011467993.1, XM_011468394.1, XM_004302782.2, XM_011470598.1, XM_011462414.1, XM_011462870.1, XM_011460468.1, XM_011460532.1, XM_004306835.2, XM_004292734.2, XM_011461437.1, XM_004288669.2, XM_004297378.2, XM_004309863.2, XM_011463324.1, XM_011462117.1, XM_011461187.1, XM_011469191.1, XM_004301401.2, XM_004307111.2, XM_011460351.1, XM_011459642.1, XM_011466431.1, XM_004307502.2, XM_011459511.1, XM_011461024.1, XM_004293937.2, XM_004300220.2, XM_011468003.1, XM_004300993.2, XM_004290283.2, XM_011461534.1, XM_004306966.2, XM_011463500.1, XM_004290492.2, XM_004301430.2, XM_011468713.1, XM_011468668.1, XM_011464218.1, XM_004304665.2, XM_004304202.2, XM_011469627.1, XM_004309894.2, XM_011470631.1, XM_011468427.1, XM_004301305.2, XM_011468150.1, XM_011470704.1, XM_011459272.1, XM_004305202.2, XM_011461257.1, XM_004291761.2, XM_011471940.1, XM_011471039.1, XM_004295492.2, XM_011461713.1, XM_004310165.2 XM_011461981.1, XM_011463835.1, XM_011468844.1, XM_011470621.1, XM_004294647.2, XM_004304396.2, XM_011464713.1, XM_011470691.1, XM_011464210.1, XM_011466850.1, XM_011466140.1, XM_004296558.2, XM_004305077.2, XM_004300754.2, XM_011463576.1, XM_011460462.1, XM_011466353.1, XM_004308002.2, XM_011466503.1, XM_004293805.2, XM_011460689.1, XM_004300747.2, XM_011461947.1, XM_011463371.1, XM_011472139.1, XM_004308454.2, XM_011459878.1, XM_011471891.1, XM_011468987.1, XM_011464082.1, XM_011461702.1, XM_011461112.1, XM_011470451.1, XM_011461005.1, XM_011460067.1, XM_011466433.1, XM_011469109.1, XM_011465464.1, XM_011471178.1, XM_011466423.1, XM_011472750.1, XM_004305301.1, XM_004305505.1, XM_011472233.1, XM_011460423.1, XM_011471197.1, XM_011459771.1, XM_004302102.2, XM_011459624.1, XM_011459618.1, XM_011464795.1, XM_004309449.2, XM_011465996.1, XM_011471424.1, XM_004297202.2, XM_011468607.1, XM_004287170.2, XM_011471069.1, XM_011463726.1, XM_011470021.1, XM_011468524.1, XM_011466924.1, XM_004307843.2, XM_011460093.1, XM_011472766.1, XM_011468447.1, XM_004297865.2, XM_004303797.2, XM_004294154.2, XM_011459009.1, XM_011471303.1, XM_011464816.1, XM_011462229.1, XM_011460974.1, XM_004300308.2, XM_011465053.1, XM_004306324.1, XM_011464604.1, XM_011461424.1, XM_011472078.1, XM_004295305.2, XM_011464622.1, XM_011468822.1, XM_011461900.1, XM_004288665.2, XM_004301265.2, XM_004308459.2, XM_011470998.1, XM_011471317.1, XM_011470520.1, XM_011471021.1, XM_011468017.1, XM_011471502.1, XM_004295149.2, XM_004289854.2, XM_011463076.1, XM_004291964.2, XM_004297786.2, XM_011465161.1, XM_004290126.2, XM_011470429.1, XM_004299618.2, XM_011463650.1, XM_011462524.1, XM_004306784.2, XM_011462054.1, XM_004302539.2, XM_011461421.1, XM_011472329.1, XM_011462755.1, XM_004307885.2, XM_011462274.1, XM_011463917.1, XM_004288653.2, XM_011460759.1, XM_011465879.1, XM_011467912.1, XM_004301047.2, XM_011464742.1, XM_004306961.2, XM_004299853.2, XM_011466327.1, XM_011472643.1, XM_011460434.1, XM_004299596.2, XM_004291003.2, XM_004297426.2, XM_004290287.2, XM_004302532.2, XM_011467176.1, XM_011463112.1, XM_004305609.2, XM_004290031.2, XM_011461805.1, XM_011467710.1, XM_011467000.1, XM_004297187.2, XM_011466648.1, XM_011465537.1, XM_004290947.2, XM_011465518.1, XM_004296105.2, XM_004299463.2, XM_011460210.1, XM_004309798.2, XM_011466633.1, XM_004306387.2, XM_004288448.2, XM_011459946.1, XM_011461791.1, XM_011470277.1, XM_011464383.1, XM_004300043.2, XM_004304480.2, XM_004291976.1, XM_011466853.1, XM_011463506.1, XM_011466787.1, XM_004290471.2, XM_004296460.2, XM_011472540.1, XM_011463922.1, XM_011466080.1, XM_011464931.1, XM_004298534.2, XM_004296872.2, XM_011461834.1, XM_011472036.1, XM_004307083.2, XM_011460931.1, XM_011470579.1, XM_011462615.1, XM_004301461.2, XM_004290688.2, XM_011461091.1, XM_011465119.1, XM_004301559.2, XM_011469973.1, XM_011471639.1, XM_011462097.1, XM_011466799.1, XM_004302217.2, XM_011469615.1, XM_004293544.2, XM_004307085.2, XM_011459111.1, XM_004298961.2, XM_011466619.1, XM_011465622.1, XM_011465516.1, XM_011459359.1, XM_011465055.1, XM_004288040.2, XM_011464566.1, XM_011460984.1, XM_011471399.1, XM_011462145.1, XM_004291720.2, XM_011466354.1, XM_011462720.1, XM_011459375.1, XM_011466558.1, XM_011460875.1, XM_011465878.1, XM_004295913.2, XM_011469536.1, XM_011463616.1, XM_004297203.2, XM_004302312.2, XM_011461664.1, XM_011459599.1, XM_011464759.1, XM_011466642.1, XM_004296472.2, XM_004302992.2, XM_004291905.2, XM_011461339.1, XM_011471268.1, XM_011466424.1, XM_004309330.2, XM_011464020.1, XM_004292932.2, XM_011468312.1, XM_011459042.1, XM_004297709.2, XM_004295473.2, XM_011469861.1, XM_004299817.2, XM_011460369.1, XM_011459457.1, XM_011462413.1, XM_011468934.1, XM_011463255.1, XM_011462862.1, XM_004309293.2, XM_004305765.2, XM_011460444.1, XM_011468438.1, XM_004291884.2, XM_004302204.2, XM_011465057.1, XM_004298113.2, XM_004298193.2, XM_011459415.1, XM_011465153.1, XM_011470496.1, XM_004299978.2, XM_004303844.2, XM_004306886.2, XM_004306834.2, XM_004295277.2, XM_004296953.2, XM_004303965.2, XM_004306706.2, XM_011463439.1, XM_004287692.2, XM_004290699.2, XM_011466993.1, XM_011460743.1, XM_011466662.1, XM_004293802.2, XM_004306197.1, XM_011460622.1, XM_011465638.1, XM_011468821.1, |
|  |  |  |  |  |  |  |  |
| **Chitosan 24 hpt up-regulated DEGs (Total sequences in DEGs set: 814, in Reference Transcriptome: 13812)** | | | | | | | |
| GO:0009765 | photosynthesis, light harvesting | P | 8.48E-10 | 2.60E-13 | 15 | 10 | XM_011463913.1, XM_011467315.1, XM_011463356.1, XM_004296111.2, XM_004297284.2, XM_011470873.1, XM_011472504.1, XM_011470681.1, XM_004301401.2, XM_004300993.2, XM_004301430.2, XM_004301305.2, XM_011464795.1, XM_004306324.1, XM_011471502.1, XM_004296105.2, XM_011470277.1, XM_011466787.1, XM_011463922.1, XM_011471399.1, XM_011463616.1, XM_011462413.1, XM_011462862.1, XM_004309293.2. |
| GO:0016168 | chlorophyll binding | F | 1.49E-03 | 2.06E-06 | 7 | 6 | XM_004301559.2, XM_004294219.2, XM_004299300.2, XM_004310031.2, XM_004304664.2, XM_004293412.2, XM_004290823.2. |
| GO:0045735 | nutrient reservoir activity | F | 2.63E-02 | 7.65E-05 | 8 | 19 | XM_011459701.1, XM_004289896.2, XM_011459702.1, XM_004292530.2, XM_004289898.2, XM_004294067.2, XM_004289897.2, XM_011459704.1. |
| GO:0009522 | photosystem I | C | 3.85E-04 | 4.72E-07 | 9 | 11 | XM_004302848.2, XM_011461118.1, XM_004294219.2, XM_004306562.2, XM_004310031.2, XM_004302108.2, XM_004304664.2, XM_004288072.2, XM_004293412.2. |
| GO:0009523 | photosystem II | C | 2.64E-03 | 4.46E-06 | 11 | 27 | psbK, XM_004302411.2, XM_004288102.2, XM_004306480.2, XM_004289855.2, XM_004294219.2, XM_004310031.2, XM_004303854.2, XM_004300527.2, XM_004303427.2, XM_004293412.2. |
| GO:0009535 | chloroplast thylakoid membrane | C | 1.27E-02 | 2.74E-05 | 21 | 118 | XM_004304335.2, XM_004302848.2, XM_011469952.1, XM_004299306.2, XM_004295077.2, XM_004307910.2, XM_004306459.2, XM_004300655.2, XM_004294219.2, XM_004306420.2, XM_004306562.2, XM_004299300.2, XM_004310031.2, XM_004300527.2, XM_011462623.1, XM_004304664.2, XM_004303852.2, XM_004288072.2, XM_004293412.2, XM_004291275.2, XM_004290823.2. |
| GO:0012511 | monolayer-surrounded lipid storage body | C | 4.25E-02 | 1.31E-04 | 4 | 2 | XM_004307729.2, XM_004292787.2, XM_004304601.2, XM_004292786.1. |
| GO:0010287 | plastoglobule | C | 4.62E-02 | 2.12E-04 | 6 | 11 | XM_004303852.2, XM_004302848.2, XM_004300655.2, XM_011460749.1, XM_004299300.2, XM_004300527.2. |

*** =** P, biological process; F, molecular function; C, cellular component.
